# Supplementary material for: Controlling cyclodextrin host-guest complexation in water with dynamic pericyclic chemistry
Source: Commun Chem. 2025 Dec 26;9:51. doi: 10.1038/s42004-025-01858-8 (PMC12847988; doi:10.1038/s42004-025-01858-8)
Supplement: Supplementary file 2 — Supporting Information [file 42004_2025_1858_MOESM2_ESM.pdf]

# Supporting Information

## Controlling cyclodextrin host-guest complexation in water with dynamic pericyclic chemistry

Marius Gaedke,[a] Anja Ramström,[a] Daisy R. S. Pooler,[a] and Fredrik Schaufelberger*[a,b]

a. KTH Royal Institute of Technology, Department of Chemistry, Teknikringen 30, 10044 Stockholm, Sweden.
b. Department of Chemistry, University of Warwick, Gibbet Hill Rd, Coventry CV4 7AL, UK.

*Corresponding author e-mail: fredrik.schaufelberger@warwick.ac.uk; fresch@kth.se;

## Table of contents

| | |
|---|---|
| S1. Abbreviations | S1 |
| S2. General experimental | S2 |
| S3. Reaction schemes | S3 |
| S4. Experimental procedures | S4 |
| S5. Reaction optimisation and kinetics | S8 |
| S6. Binding constant determinations | S13 |
| S7. Additional switching experimental data | S20 |
| S8. Fluorescence spectroscopy | S30 |
| S9. Molecular modelling | S32 |
| S10. NMR Spectra | S34 |
| S11. Mass Spectra | S40 |
| S12. Supplementary information references | S43 |

## S1. ABBREVIATIONS

Abbreviations: CD cyclodextrin; COSY correlation spectroscopy; CREST conformer rotamer ensemble sampling tool; Cys cysteine; DCM dichloromethane; DEPT distortionless enhancement by polarization transfer; DMF *N*,*N*-dimethylformamide; DMSO dimethylsulfoxide; DTT dithiothreitol; GSH glutathione; HEPES 4-(2-hydroxyethyl)-1-piperazineethanesulfonic acid; HMBC heteronuclear multiple bond correlation; HPLC high-performance liquid chromatography; HRMS high resolution mass spectrometry; HSQC heteronuclear single quantum coherence; LCST lower critical solution temperature; MeCN acetonitrile; NMR nuclear magnetic resonance; o.n. overnight; PE petroleum ether (40-60 °C bp.); RT room temperature; THF tetrahydrofuran; TLC thin layer chromatography.

;

## S2. GENERAL EXPERIMENTAL

All reagents and solvents were obtained from commercial sources and used without further purification unless stated otherwise. Reactions were carried out in anhydrous solvents and under an $N_2$ atmosphere. Anhydrous solvents were obtained by passing the solvent through an activated alumina column in a Glass Contour solvent dispensing system and stored over molecular sieves. Compounds **S1**[1], **S4**[2], triethylene glycol methylether tosylate[3] and β-CD-$N_3$[4] were synthesised according to literature procedures.

$^1$H NMR spectra were recorded on a Bruker Avance DMX 500 MHz NMR spectrometer and a Bruker Ascend 400 spectrometer (400 MHz). Chemical shifts are reported in parts per million (ppm) from high to low frequency using the residual solvent peak as the internal reference ($CDCl_3$ = 7.26 ppm). All $^1$H resonances are reported to the nearest 0.01 ppm. The multiplicity of $^1$H signals are indicated as: s = singlet; d = doublet; t = triplet; q = quartet; m = multiplet; br = broad; app = apparent; or combinations thereof. Coupling constants (*J*) are quoted in Hz and reported to the nearest 0.1 Hz. Where appropriate, averages of the signals from peaks displaying multiplicity were used to calculate the value of the coupling constant. $^{13}$C NMR spectra were recorded on the same spectrometers with the central resonance of the solvent peak as the internal reference ($CDCl_3$ = 77.16 ppm). All $^{13}$C resonances are reported to the nearest 0.01 ppm. For new compounds, DEPT, COSY, HSQC and HMBC experiments were used to aid spectral assignment. Computational models were optimized using HF-3c implemented in the ORCA software version 5.0.4.

Flash column chromatography was carried out using Silica 60 Å (particle size 40–63 µm, Merck, Sweden) as the stationary phase. TLC was performed on precoated silica gel plates (0.25 mm thick, 60 $F_{254}$, Merck, Germany) and visualized using both short and long wave ultraviolet light in combination with standard laboratory stains (basic potassium permanganate, acidic ammonium molybdate). High-resolution mass spectrometry was performed on a 1260 Infinity II HPLC (Poroshell 120 EC-C18 2.1x50mm, 1.9 µm column using 0.6 mL/min flow) connected to a MS-QTOF 6530C instrument (Agilent). The high-resolution mass spectra of Diels Alder adducts were collected from reaction mixtures using reversed phase HPLC or flow injection analysis:

UV/Vis spectra were recorded with a PerkinElmer Lambda 750 spectrometer. Fluorescence spectra were recorded with a Varian Cary Eclipse spectrometer. Solvents with HPLC grade and Suprasil glass cuvettes with a path-length of 1 cm were used. Fluorescence was measured at 100 µM concentration in either water with NaOH or HEPES buffer. ($\lambda_{ex}$ = 360 nm, Slit 2.5, 380-600 nm).

# S3. REACTIONS SCHEMES

## S3.1. Synthesis of anthracenes

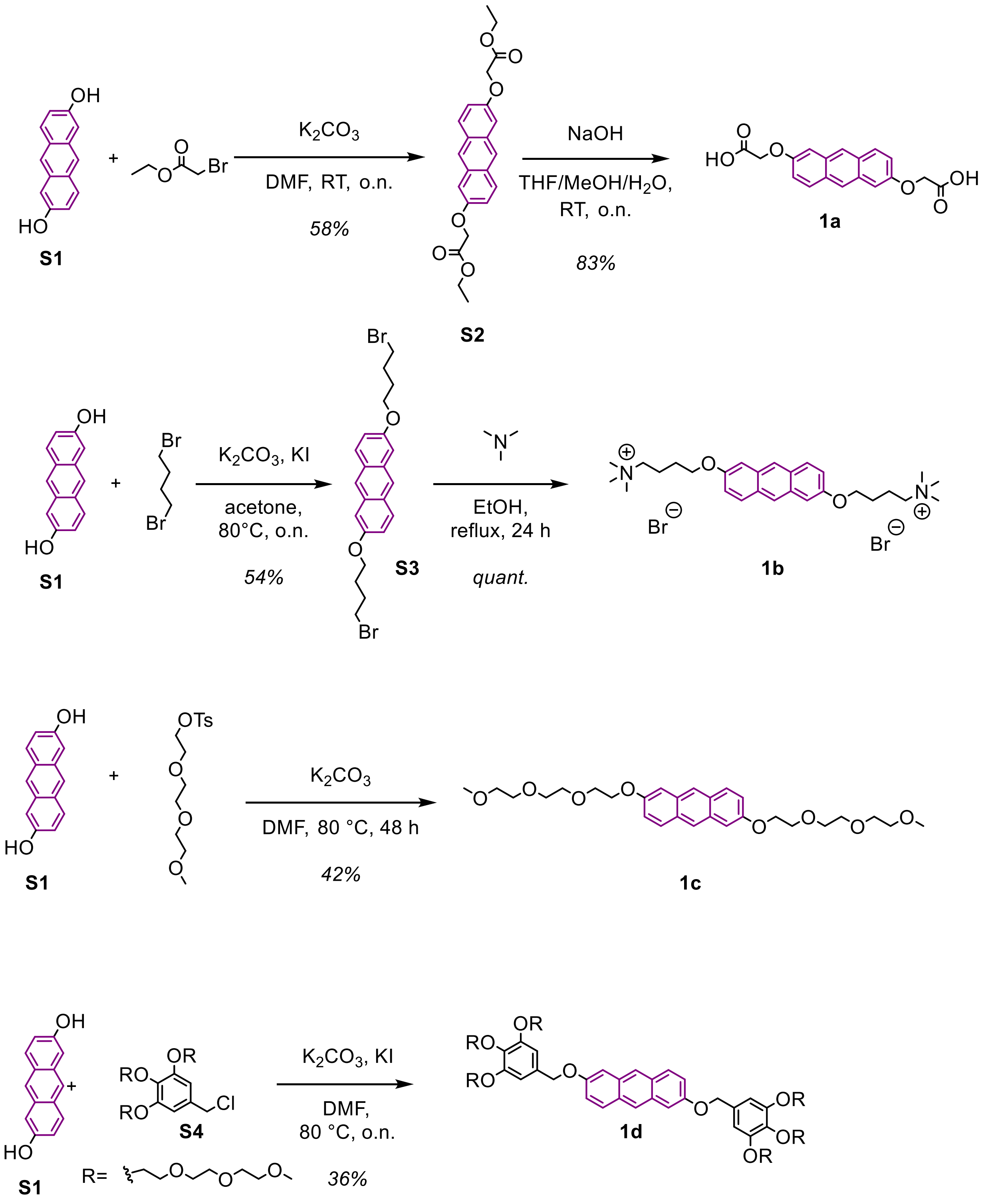


**Scheme S1.** Procedure for anthracenes **1a-d**.

# S4. EXPERIMENTAL PROCEDURES

## S4.1. Synthetic procedures and characterisation details

### S2

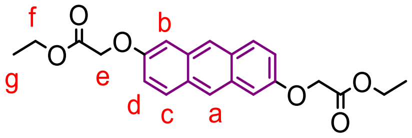

A solution of 631 mg (3.00 mmol, 1.0 equiv.) anthracene-2,6-diol **S1**, 1.20 g (7.2 mmol, 2.4 equiv.) ethyl 2-bromoacetate and 1.66 g (12.0 mmol, 4.0 equiv.) $K_2CO_3$, in dry DMF (30 mL) was stirred at RT under $N_2$ atmosphere overnight. Afterwards the solvent was removed under reduced pressure, the residue was re-dissolved in $CH_2Cl_2$ and then washed with water and brine. The organic phase was dried with $MgSO_4$, filtered and concentrated. The crude product was purified by column chromatography ($SiO_2$, $CH_2Cl_2$ $R_f$ ~ 0.5) to obtain the product **S2** as a yellowish solid (665 mg, 1.74 mmol, 58 %).

**$^1$H NMR** (500 MHz, $CDCl_3$): $\delta$ = 8.20 (s, 2H, a), 7.87 (d, $J$ = 9.2 Hz, 2H, c), 7.28 – 7.22 (m, 2H, d), 7.11 (d, $J$ = 2.4 Hz, 2H, b), 4.77 (s, 4H, e), 4.32 (q, $J$ = 7.1 Hz, 4H, f), 1.32 (t, $J$ = 7.1 Hz, 6H, g) ppm.
**$^{13}$C NMR** (126 MHz, $CDCl_3$): $\delta$ = 168.95, 155.03, 131.22, 129.80, 129.12, 124.77, 120.55, 105.36, 65.58, 61.62, 14.35 ppm.
**HRMS** (MeCN): *m/z* calcd. for [$C_{22}H_{22}O_6$]: 383.1495 $[M+H]^+$, found: 383.1481; 405.1314 $[M+Na]^+$, found: 405.1304.

### 1a

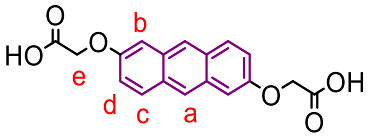

A solution of 191 mg (0.5 mmol, 1.0 equiv.) anthracene ester **S2** and 680 mg (17 mmol, 34 equiv.) NaOH (in 17 mL $H_2O$) was dissolved in a mixture of THF (15 mL) and MeOH (15 mL), and subsequently stirred at RT overnight. Afterwards, the mixture was concentrated under reduced pressure to a volume of around 10 mL. Hydrochloric acid (4 mL, 6 M aq.) was added and the mix was left to precipitate overnight. The product was isolated via vacuum filtration, washed with $H_2O$ and dried in vacuo. The product **1a** was isolated as yellowish solid (136 mg, 0.42 mmol, 83 %).
**$^1$H NMR** (500 MHz, DMSO-d6): $\delta$ = 13.09 ($s_{br}$, 2H, COOH), 8.33 (s, 2H, a), 7.96 (d, $J$ = 9.2 Hz, 2H, c), 7.28 (d, $J$ = 1.6 Hz, 2H, d), 7.21 (dd, $J$ = 9.1, 2.1 Hz, 2H, b), 4.82 (s, 4H, e) ppm.
**$^{13}$C NMR** (126 MHz, DMSO-d6): $\delta$ = 170.03, 154.54, 130.68, 129.36, 128.32, 124.26, 120.25, 105.12, 64.56 ppm.
**HRMS** (MeCN): *m/z* calcd. for [$C_{18}H_{14}O_6$]: 327.0869 $[M+H]^+$, found: 327.0875; 349.0688 $[M+Na]^+$, found: 349.0698.

**S3**

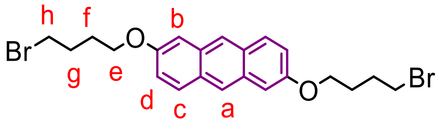


A solution of 200 mg (0.96 mmol, 1.0 equiv.) anthracene-2,6-diol **S1**, 2.06 g (9.6 mmol, 1.13 mL, 10 equiv.) 1,4-dibromobutane, 293 mg (2.1 mmol, 2.2 equiv.) $K_2CO_3$ and 17 mg (0.1 mmol, 0.1 equiv.) KI, in dry acetone (80 mL), was stirred at 50 °C in an $N_2$ atmosphere overnight. Afterwards the solvent was removed under reduced pressure, the residue was re-dissolved in $CH_2Cl_2$ and then washed with water and brine. The organic phase was dried with $MgSO_4$, filtered and concentrated. The crude product was purified by column chromatography ($SiO_2$, $CH_2Cl_2$/PE 1:1, $R_f$ ~ 0.5) to obtain the product **S3** as a yellowish solid (249 mg, 0.52 mmol, 54 %).

**$^1$H NMR** (500 MHz, $CD_2Cl_2$): $\delta$ = 8.20 (s, 2H, a), 7.85 (d, $J$ = 9.1 Hz, 2H, c), 7.19 (d, $J$ = 2.3 Hz, 2H, d), 7.14 (dd, $J$ = 9.1; 2.4 Hz, 2H, b), 4.15 (t, $J$ = 6.1 Hz, 4H, e), 3.55 (t, $J$ = 6.7 Hz, 4H, h), 2.13 (dt, $J$ = 11.8, 6.8 Hz, 4H, g), 2.02 (dt, J = 15.3, 6.1 Hz, 4H, f) ppm.

**$^{13}$C NMR** (126 MHz, $CD_2Cl_2$): $\delta$ = 156.42, 131.83, 129.72, 129.28, 124.69, 121.21, 105.13, 67.49, 34.33, 30.26, 28.48 ppm.

**HRMS** (MeCN): *m/z* calcd. for [$C_{22}H_{24}O_2Br_2$]: 480.0123 [M-e]$^+$, found: 480.0117.

**1b**

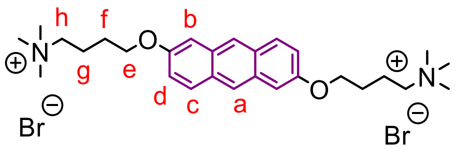


A solution of 60 mg (0.13 mmol, 1.0 equiv.) dibromide **S3**, 10 mL of 4.2 M in EtOH (42 mmol, 323 equiv.) $NMe_3$ refluxed in an $N_2$ atmosphere for 24 h. Afterwards the solvent was removed under reduced pressure to obtain the product **1b** as a yellowish solid (78 mg, 0.13 mmol, quant.).

**$^1$H NMR** (500 MHz, $D_2O$+1% MeOD-d4): $\delta$ = 8.21 (s, 2H, a), 7.89 (d, $J$ = 9.0 Hz, 2H, c), 7.26 (s, 2H, d), 7.15 (d, $J$ = 8.3; 2H, b), 4.13 (t, $J$ = 5.2 Hz, 4H, e), 3.38 – 3.31 (m, 4H, h), 3.07 (m ,18H, NMe), 1.98 – 1.88 (m, 4H, g), 1.88 – 1.80 (m, 4H, f) ppm.

**$^{13}$C NMR** (126 MHz, $D_2O$+1% MeOD-d4): $\delta$ = 156.01, 131.89, 130.52, 129.45, 125.36, 121.23, 106.28, 68.16, 67.15, 53.78. 53.75, 53.72, 26.14, 20.40 ppm.

**HRMS** (MeCN): *m/z* calcd. for [$C_{28}H_{42}O_2N_2Br_2$]: 219.1618 [M-2Br]$^{2+}$, found: 219.1629.

**1c**

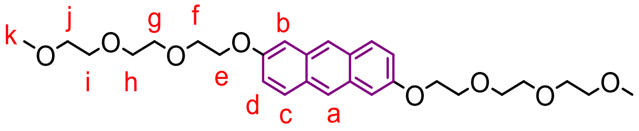

A solution of 200 mg (0.95 mmol, 1.0 equiv.) anthracene-2,6-diol **S1**, 758 mg (2.38 mmol, 2.5 equiv.) triethylene glycol methylether tosylate and 526 mg (3.81 mmol, 4.0 equiv.) $K_2CO_3$, in dry DMF (50 mL) were heated to 80 °C in an argon atmosphere for 48 h. Afterwards the solvent was removed under reduced pressure, the residue was re-dissolved in $CH_2Cl_2$ and then washed with water and brine. The organic phase was dried with $MgSO_4$, filtered and concentrated. The crude product was purified by column chromatography ($SiO_2$, EtOAc $R_f$ ~ 0.4) to obtain the product **1c** as a yellowish oil (202 mg, 0.4 mmol, 42 %).

**$^1$H NMR** (400 MHz, $CDCl_3$): $\delta$ = 8.17 (s, 2H, a), 7.86 – 7.80 (m, 2H, c), 7.20 – 7.15 (m, 4H, b,d), 4.33 – 4.24 (m, 4H, e), 3.99 – 3.92 (m, 4H, f), 3.82 – 3.76 (m, 4H, g), 3.75 – 3.64 (m, 8H, I,h), 3.56 (dd, $J$ = 5.7, 3.7 Hz, 4H, j), 3.38 (s, 6H, k) ppm.
**$^{13}$C NMR** (101 MHz, $CDCl_3$): $\delta$ = 155.83, 131.33, 129.36, 128.89, 124.42, 120.90, 104.93, 72.10, 71.04, 70.86, 70.76, 69.91, 67.50, 59.20 ppm.
**HRMS** (MeCN): *m/z* calcd. for $[C_{28}H_{38}O_8]$: 525.2464 $[M+Na]^+$, found: 525.2461.

**1d**

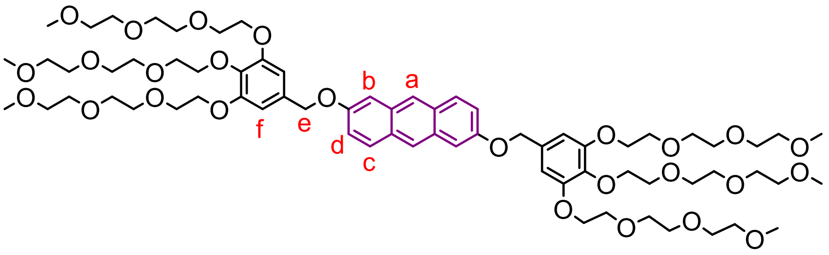

A solution of 8 mg (0.04 mmol, 1.0 equiv.) anthracene-2,6-diol **S1**, 50 mg (0.08 mmol, 2.2 equiv.) **S4**, 13 mg (0.09 mmol, 2.5 equiv.) $K_2CO_3$ and 1.2 mg (0.01 mmol, 0.1 equiv.) KI, in dry DMF (20 mL) were stirred at 80 °C in an $N_2$ atmosphere overnight. Afterwards the solvent was removed under reduced pressure and the residue was dissolved in $CH_2Cl_2$ and then washed with water and brine. The organic phase was dried with $MgSO_4$ and the solvent removed again. The crude product was purified by column chromatography followed by preparative TLC ($SiO_2$, $CH_2Cl_2$/MeOH 5%, $R_f$ ~ 0.2) to obtain the product **1d** as a brown oil (20 mg, 0.15 mmol, 36%). The product exhibits an LCST in aqueous solution between 55 and 60 °C.
**$^1$H NMR** (500 MHz, MeOD): $\delta$ = 8.19 (s, 2H, a), 7.85 (d, $J$ = 9.2 Hz, 2H, c), 7.31 (d, $J$ = 1.9 Hz, 2H, d), 7.19 (dd, $J$ = 9.2; 1.9 Hz, 2H, b), 6.84 (s, 2H, f), 5.13 (s, 4H, e), 4.19 – 4.16 (m, 8H, Ar-O-C<u>H</u>$_2$-CH$_2$-O), 4.16 – 4.13 (m, 4H, Ar-O-C<u>H</u>$_2$-CH$_2$-O), 3.84 – 3.80 (m, 8H, Ar-O-CH$_2$-C<u>H</u>$_2$-O), 3.80 – 3.77 (m, 4H, Ar-O-CH$_2$-C<u>H</u>$_2$-O), 3.71 – 3.65 (m, 8H, O-CH$_2$-CH$_2$-O), 3.63 – 3.56 (m, 28H, O-CH$_2$-CH$_2$-O), 3.53 – 3.46 (m, 12H, C<u>H</u>$_2$-O-Me), 3.31 (s, 18H, OMe) ppm.

$^{13}$**C NMR** (126 MHz, MeOD): *δ* = 156.91, 154.06, 139.05, 134.39, 132.75, 130.40, 130.31, 130.26, 125.44, 121.72, 108.24, 106.68, 73.51, 72.99, 72.96, 71.75, 71.74, 71.65, 71.63, 71.48, 71.38, 70.96, 70.88, 70.05, 59.09 ppm.

**HRMS** (MeCN): *m/z* calcd. for [$C_{70}H_{106}O_{26}$]: 1380.7316 [$M+NH_4$]$^+$, found: 1380.7330.

## S4.2. General experimental procedures

### Representative protocol for forward Diels-Alder reaction

In an NMR tube with deuterated solvent (1 mL, $D_2O$ or $CDCl_3$), anthracene was added (1 mM final concentration). Dienophile (10 equiv.) was added and the tube was left at the given reaction temperature in the dark under ambient atmosphere. The reaction was monitored over time using $^1$H NMR spectroscopy and conversion was calculated by observing the ratio between product protons and the starting anthracene (most often central 9,10-anthracene protons at ~8.3 ppm). In all cases, clean conversion was observed. The NMR tube with the reaction was kept at the temperature given in Table S1 for the full duration of the experiment.

### Retro-Diels-Alder reaction in DMSO-$d_6$

Anthracene was dissolved in DMSO-$d_6$ (1 mM) at room temperature under air in an NMR tube. TCNE (10 equiv.) was added in one batch and the tube was shaken once. The forward reaction was confirmed to be complete by the time a first $^1$H-NMR spectrum could be recorded (<5 min). Retro reaction occurred spontaneously over 48-72 h, and conversion was monitored with NMR spectroscopy.

### Retro-Diels-Alder reaction in water

Reaction was initiated by addition of dienophile scavenger (10 equiv.) to a solution of the Diels-Alder adduct in $D_2O$ (1 mM). The reaction proceeded spontaneously at ambient temperature and atmosphere without stirring. For fluorescence measurements, a similar protocol was followed, but an aliquot was taken from the reaction mixture and diluted with $H_2O$ to 100 μM before measurement.

### Host-guest titrations

To a solution of anthracene **1a** (2 mM) in $D_2O$ (with 8 mM NaOD to ensure deprotonation) in an NMR tube was added consecutive aliquots of cyclodextrin (10 mM) solution, and in between each addition $^1$H-NMR spectra were recorded. The data was fitted using the anthracene protons at 8.3, 8.0, 7.3 and 4.6 ppm with a 1:1 binding model with bindfit (http://supramolecular.org).[5,6]

# S5. REACTION OPTIMISATION AND KINETICS

## S5.1. Diels-Alder reaction scope with anthracene 1a

**Table S1.** Screening data of DA reaction between **1a** (1 mM) with various dienophiles (10 equiv.) determined by $^{1}H$ NMR (400 MHz, $CDCl_3$ or $D_2O$ with 8 mM NaOD, 298 or 315 K).

| Dienophile | T (°C) | $t_{½}$ ($CDCl_3$) | $t_{½}$, ($D_2O$) |
|---|---|---|---|
| 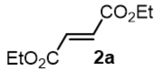 2a | 20 | 4 d | 14 d (20 equiv.) |
| 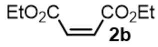 2b | 20 | > 7 d | No reaction |
| 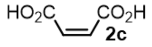 2c | 20 | No reaction | No reaction |
| 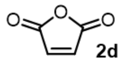 2d | 20 | 7 d | decomposition |
| 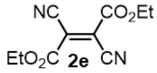 2e | 20 | >7 d | 10 h (heterogeneous) |
| | 37 | 4 d | N/A |
| 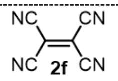 2f | 20 | < 1 min | < 1 min |
| | 37 | < 1 min | < 1 min |
| 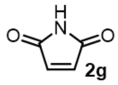 2g | 20 | 4 days | 5 h |
| | 37 | N/A | 1.5 h |
| 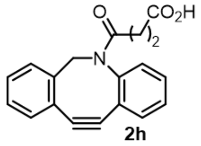 2h | 50 | - | No reaction |
| 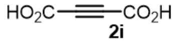 2i | 50 | - | No reaction |
| 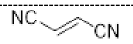 | 20 | >7 days | decomposition |
| 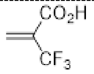 | 20 | No reaction | No reaction |
| 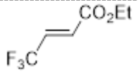 | 20 | No reaction | No reaction |

## S5.2. Kinetic data

### S.5.2.1 Eyring analysis of Diels-Alder adduct formation

For the activation parameter analysis from the data in Figure 3 (main manuscript), an Eyring plot was constructed as shown in Figure S1. The thermodynamic parameters were obtained by fitting the linearized form of the Eyring equation to the obtained data, yielding the activation parameters $\Delta H^{\ddagger}$ = +11.6 kcal $mol^{-1}$ and $\Delta S^{\ddagger}$ = -39.6 cal $K^{-1}$ $mol^{-1}$, with an overall barrier $\Delta G^{\ddagger}$ = 23.2 kcal $mol^{-1}$.

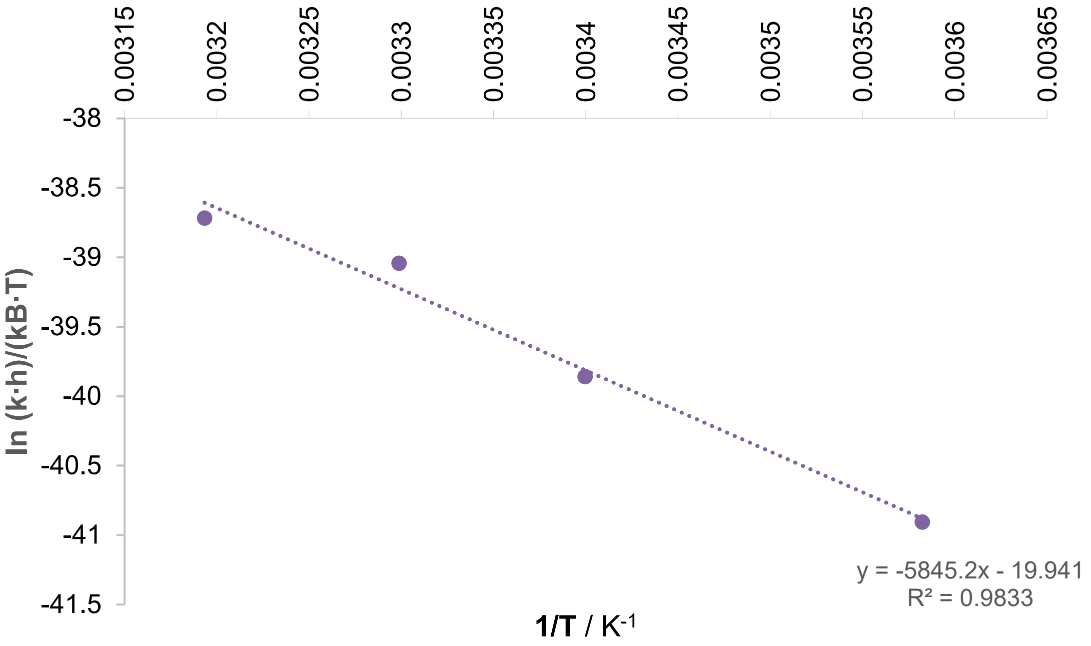


**Figure S1.** Eyring plot for the reaction of **1a** (2 mM) with maleimide **2g** (10 equiv.).

To confirm that this Diels-Alder reaction was fully under pseudo-first order conditions (with 10 equiv. of maleimide **2g**), analysis to confirm these assumptions was conducted. The measurements were repeated at 20 °C with 10, 15, 20 and 33 equiv. of **2g** (Figure S2). The observed reaction rate followed the linear relationship $k_{obs} = k \cdot [\mathbf{2g}]_0$, confirming the pseudo-first order assumption.

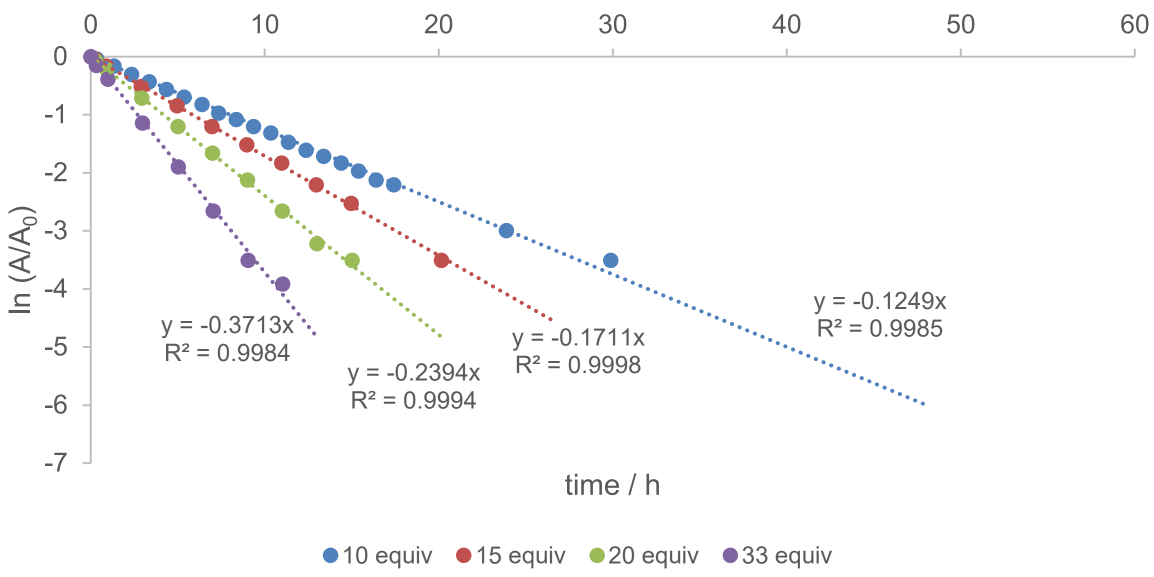


**Figure S2.** Comparison of reaction rates for **1a** (2 mM) with different equivalents of Maleimide **2g** determined by $^{1}$H NMR (400 MHz, $D_2O$ with NaOD, 298 K).

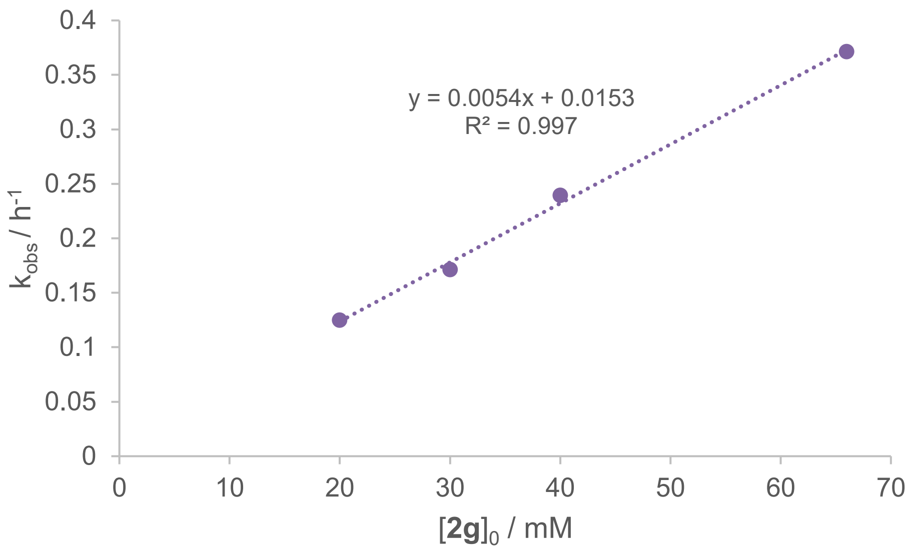


**Figure S3.** Observed reaction rates in relation to the concentration of maleimide **2g**. Note that the non-zero intercept indicates that the reaction is reversible under these conditions (298 K).

### S.5.2.2. Other kinetic traces

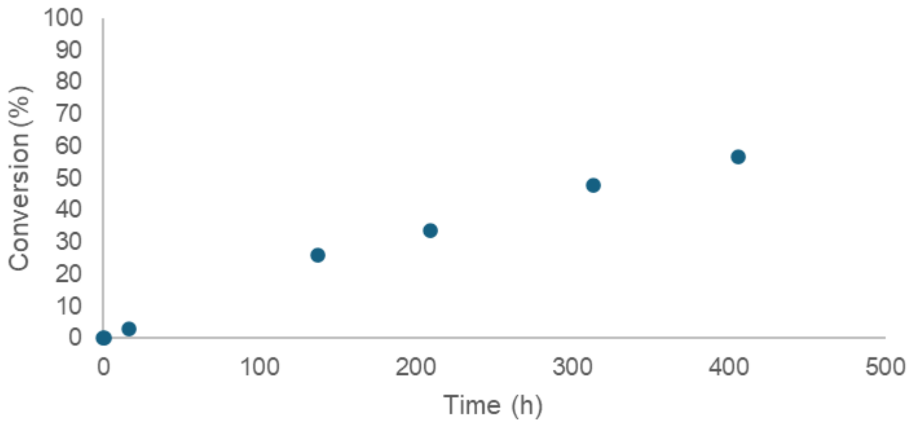


**Figure S4.** Initial rate data for the reaction of anthracene **1a** (1 mM) with activated alkene **2a** (20 mM) in $D_2O$ (with 8 mM NaOD).

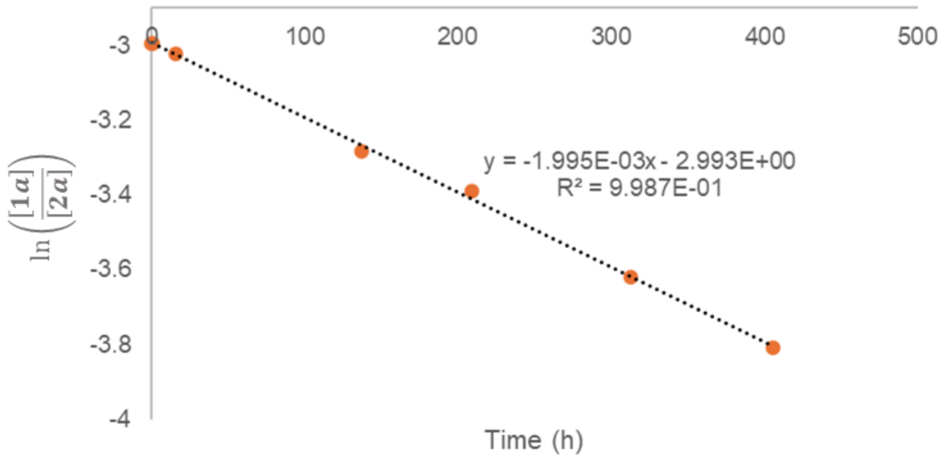


**Figure S5.** Plot of rate data using pseudo-first order reaction model for reaction between anthracene **1a** (1 mM) and activated alkene **2a** (20 mM) as per Figure S4.

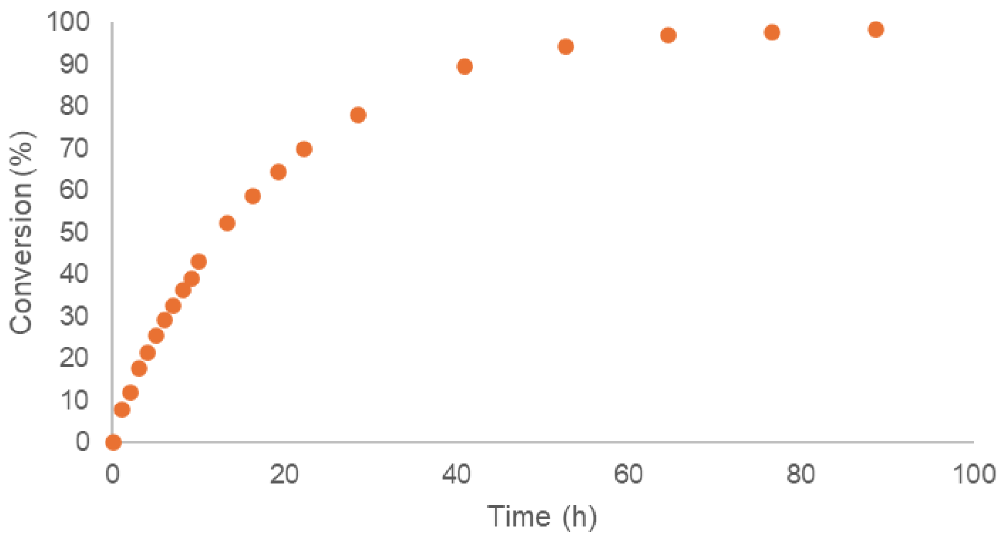


**Figure S6.** Initial rate data for the reaction of anthracene **1a** (1 mM) with maleimide **2g** (8.1 mM) in $D_2O$ (with 8 mM NaOD).

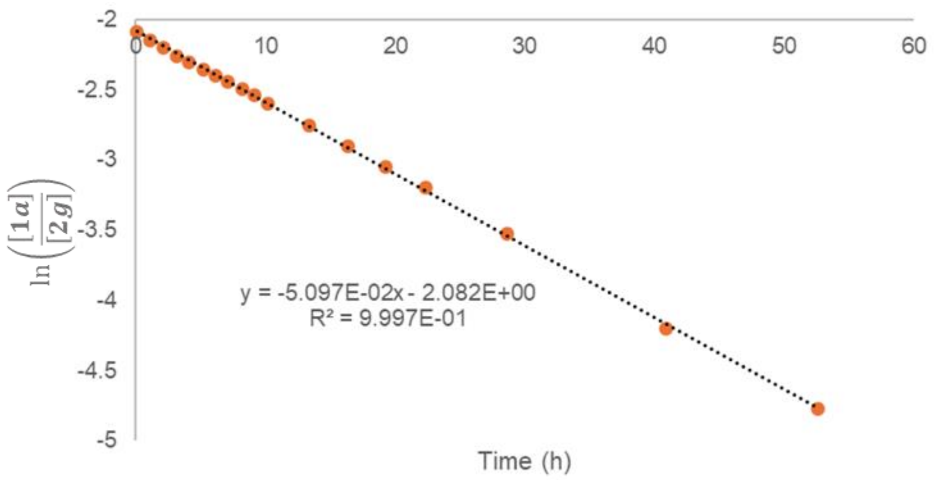


**Figure S7.** Plot of rate data using pseudo-first order reaction model for reaction between anthracene **1a** (1 mM) and maleimide **2g** (20 mM) as per Figure S6.

## S6. BINDING CONSTANT DETERMINATIONS

## 1a + α-CD

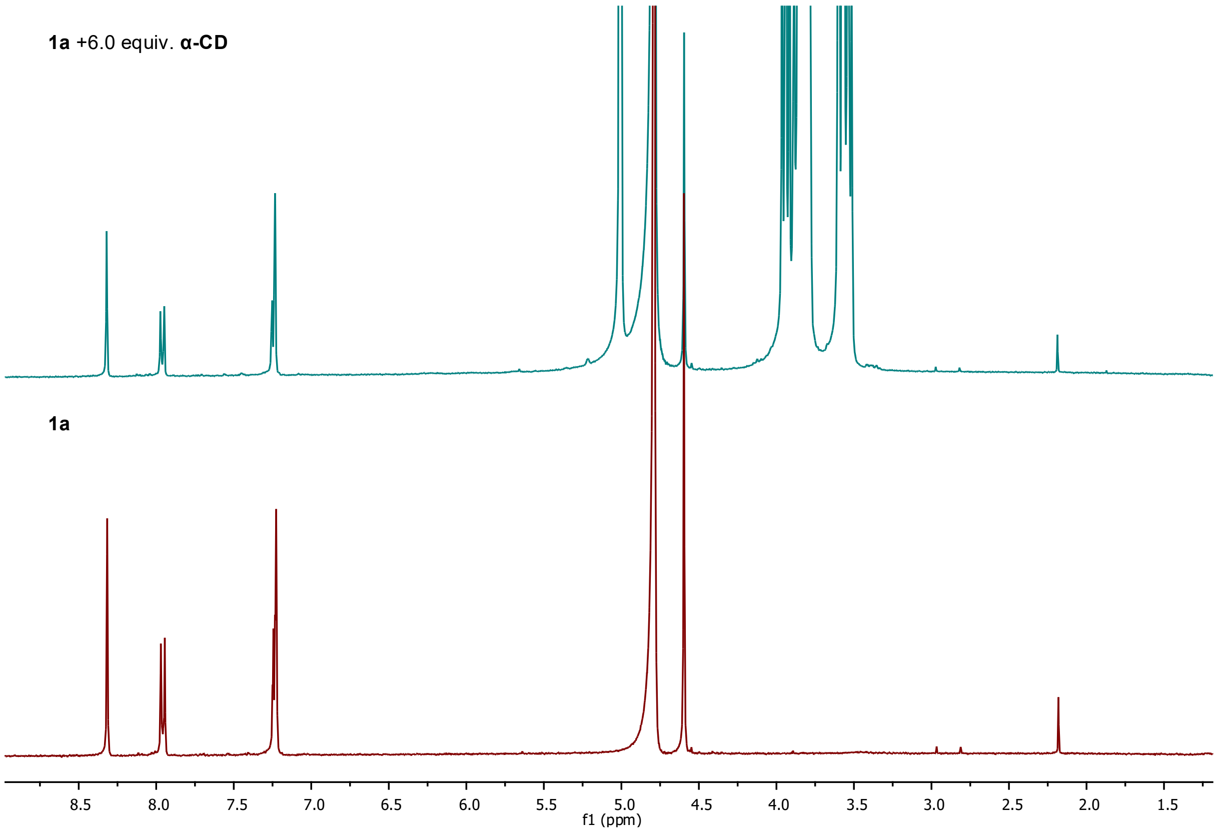


**Figure S8.** $^1$H NMR spectra (400 MHz, $D_2O$ with NaOD, 298 K) of **1a** (2 mM, bottom) and **1a** + 6.0 equiv. α-CD (top).

## 1a + β-CD

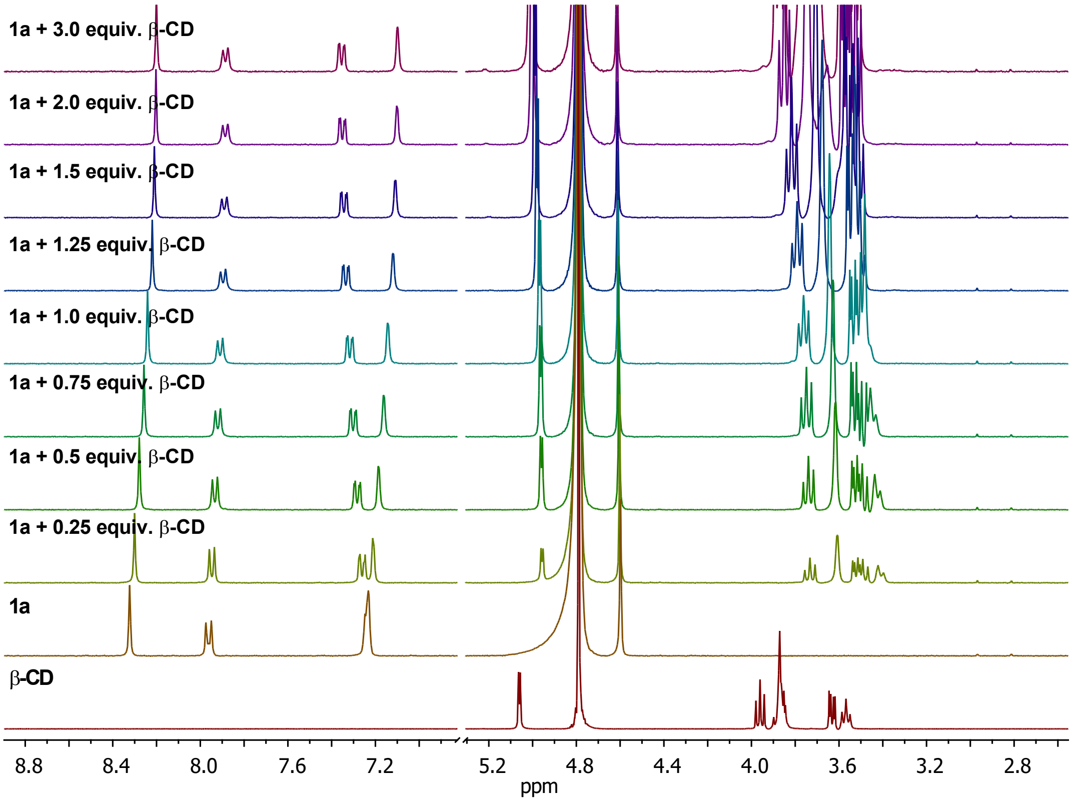


**Figure S9.** Titration of **1a** (2 mM) with increasing amounts of b-CD (bottom to top) $^1$H NMR spectrum (400 MHz, $D_2O$ with NaOD, 298 K). The binding isotherms of goodness-of-fit for this experiment can be accessed at http://app.supramolecular.org/bindfit/view/3700ef84-84be-46e5-bef1-3d090ca3886a. This experiment was performed in triplicate to determine the reproducibility of the procedure, which yielded values of 3741.8 $M^{-1}$, 3587.3 $M^{-1}$ and 3498.7 $M^{-1}$, or a mean of $(3.61 \pm 0.12) \cdot 10^3$ $M^{-1}$.

# 1a + β-CD-OMe(10-12)

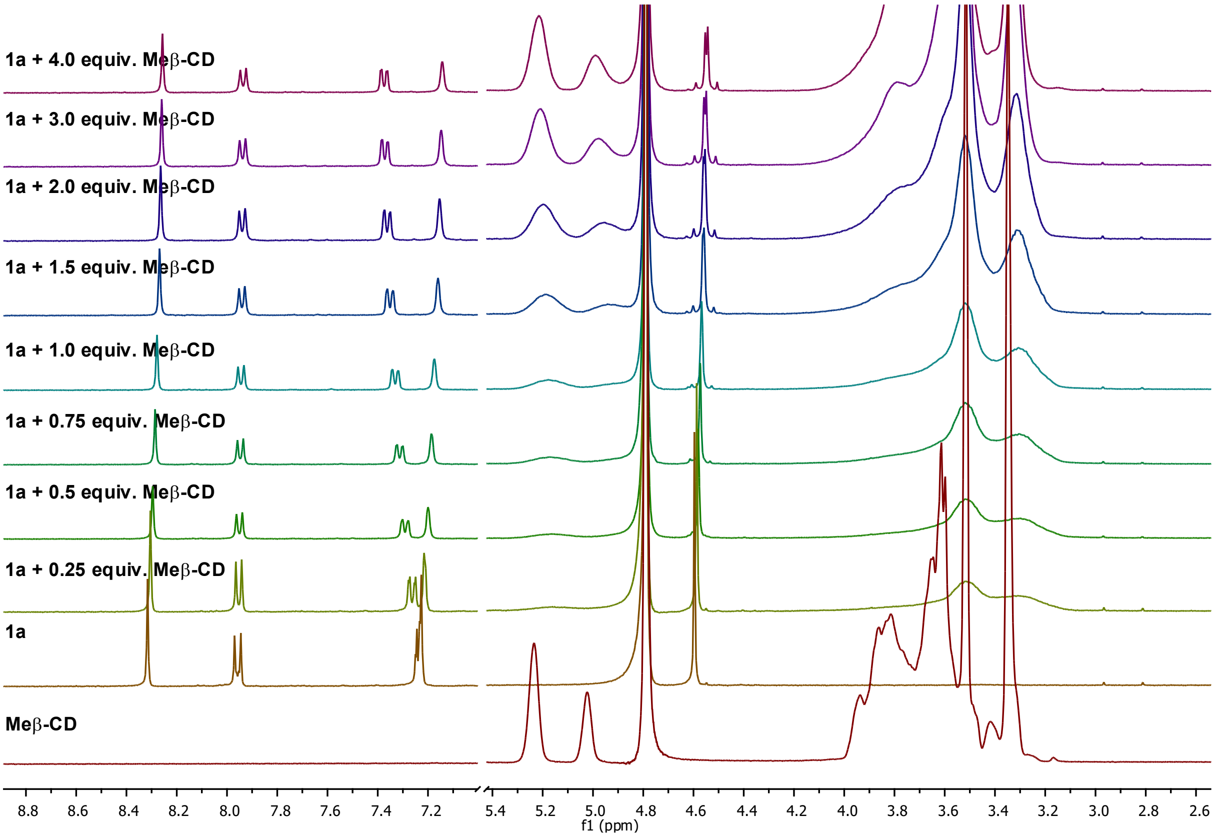


**Figure S10.** Titration of **1a** (2 mM) with increasing amounts of β-CD-OMe$_{(10\text{-}12)}$ (bottom to top) $^1$H NMR spectrum (400 MHz, $D_2O$ with NaOD, 298 K). The binding isotherms of goodness-of-fit for this experiment can be accessed at http://app.supramolecular.org/bindfit/view/0ce101d6-5a6f-4869-b79a-f71c47d0a65e.

## 1a + β-CD-N3

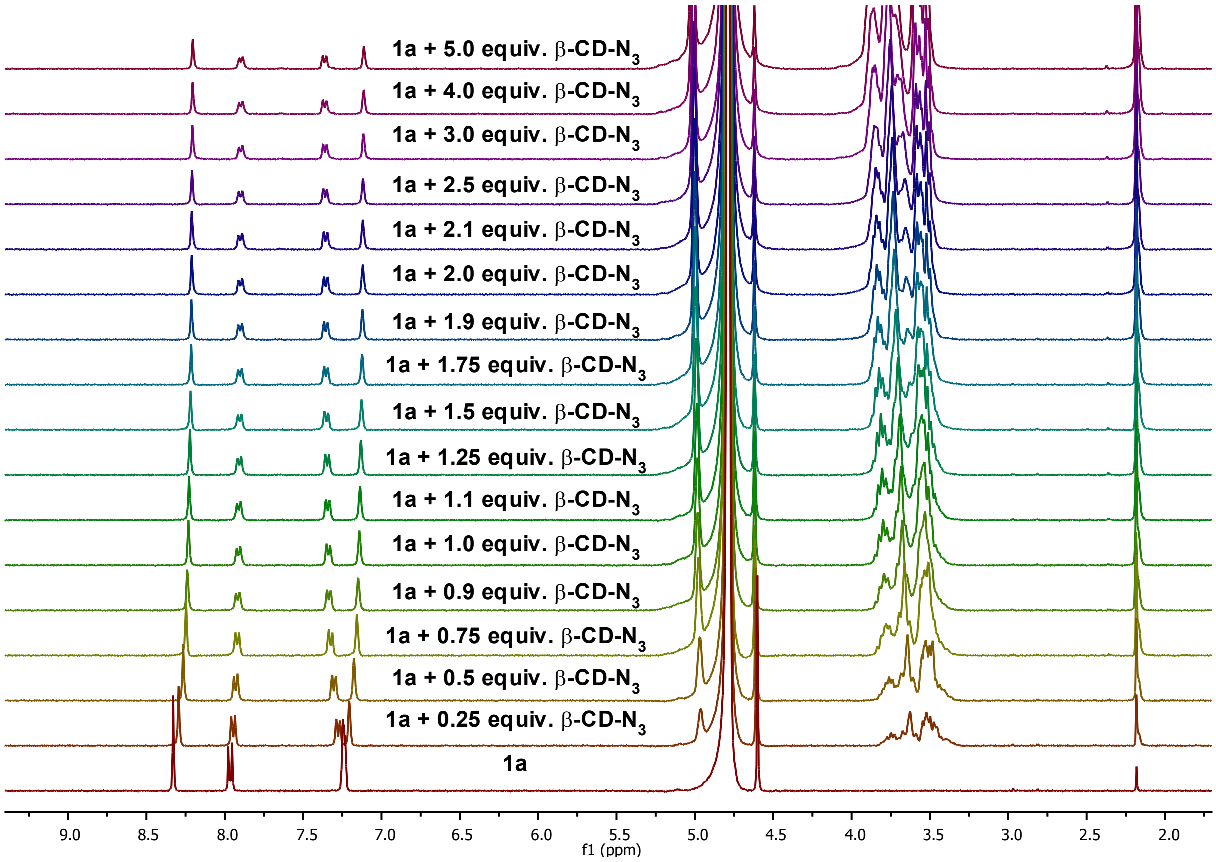


**Figure S11.** Titration of **1a** (2 mM) with increasing amounts of $N_3$-β-CD (bottom to top) $^1$H NMR spectrum (400 MHz, $D_2O$ with NaOD, 298 K). The binding isotherms of goodness-of-fit for this experiment can be accessed at http://app.supramolecular.org/bindfit/view/298d553b-3c8e-46f2-be29-26253465df9d.

# 1a + γ-CD

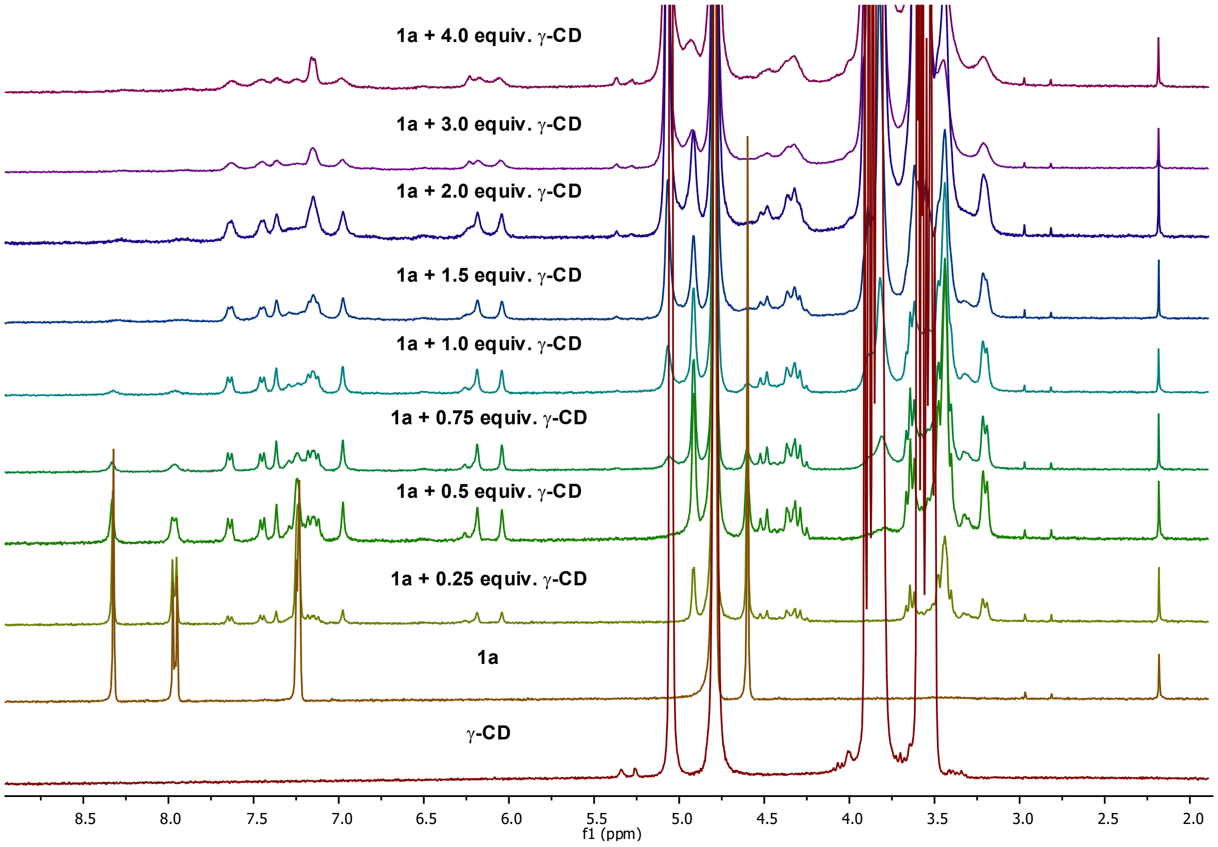


**Figure S12.** Titration of **1a** (2 mM) with increasing amounts of γ-CD (bottom to top) $^1$H NMR spectrum (400 MHz, $D_2O$ with NaOD, 298 K). Both 1:1 and 1:2 binding mode is observed, along with low amount of dimerisation of anthracenes within the cavity.[7]

## 1b + β-CD

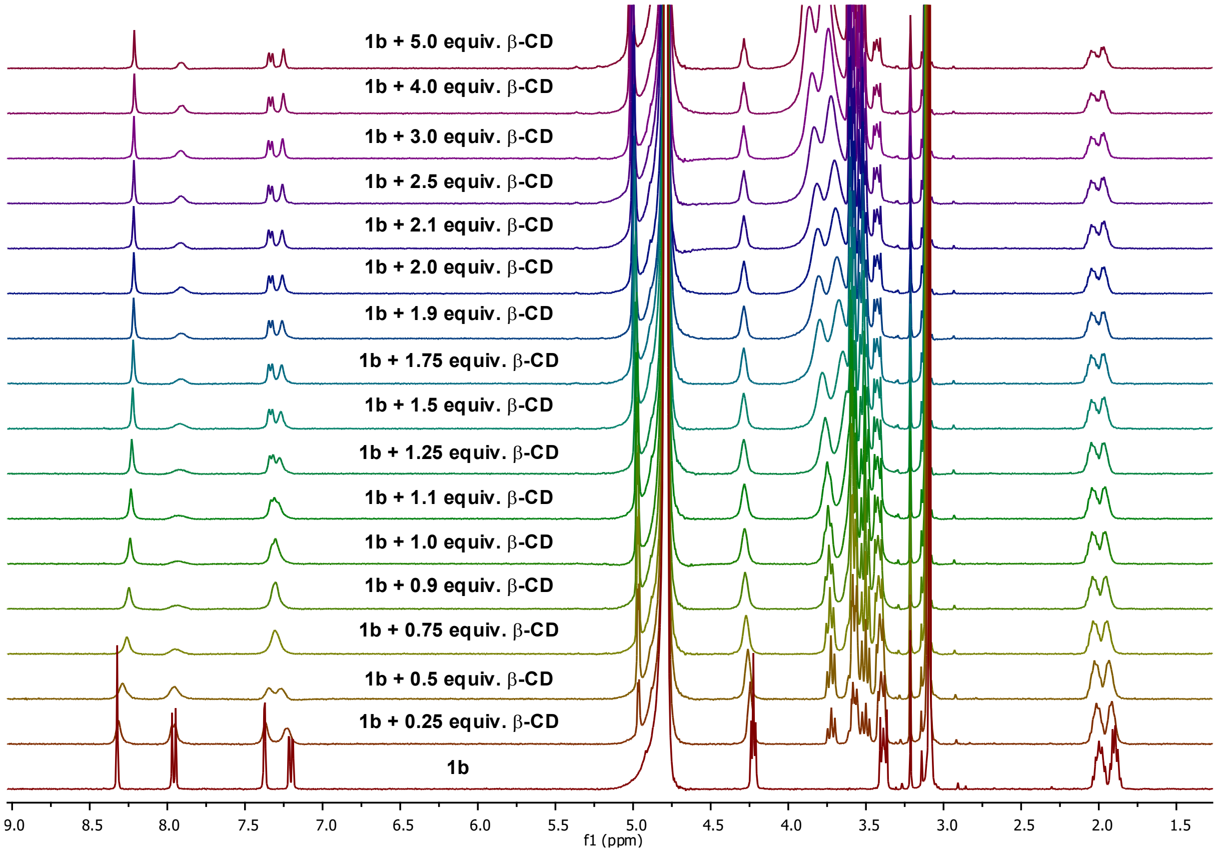


**Figure S13.** Titration of **1b** (2 mM) with increasing amounts of β-CD (bottom to top) $^{1}$H NMR spectrum (400 MHz, $D_2O$ with NaOD, 298 K). The binding isotherms of goodness-of-fit for this experiment can be accessed at http://app.supramolecular.org/bindfit/view/37de052f-2c10-4177-8ea8-d8505cc33a2b.

## 1d + β-CD

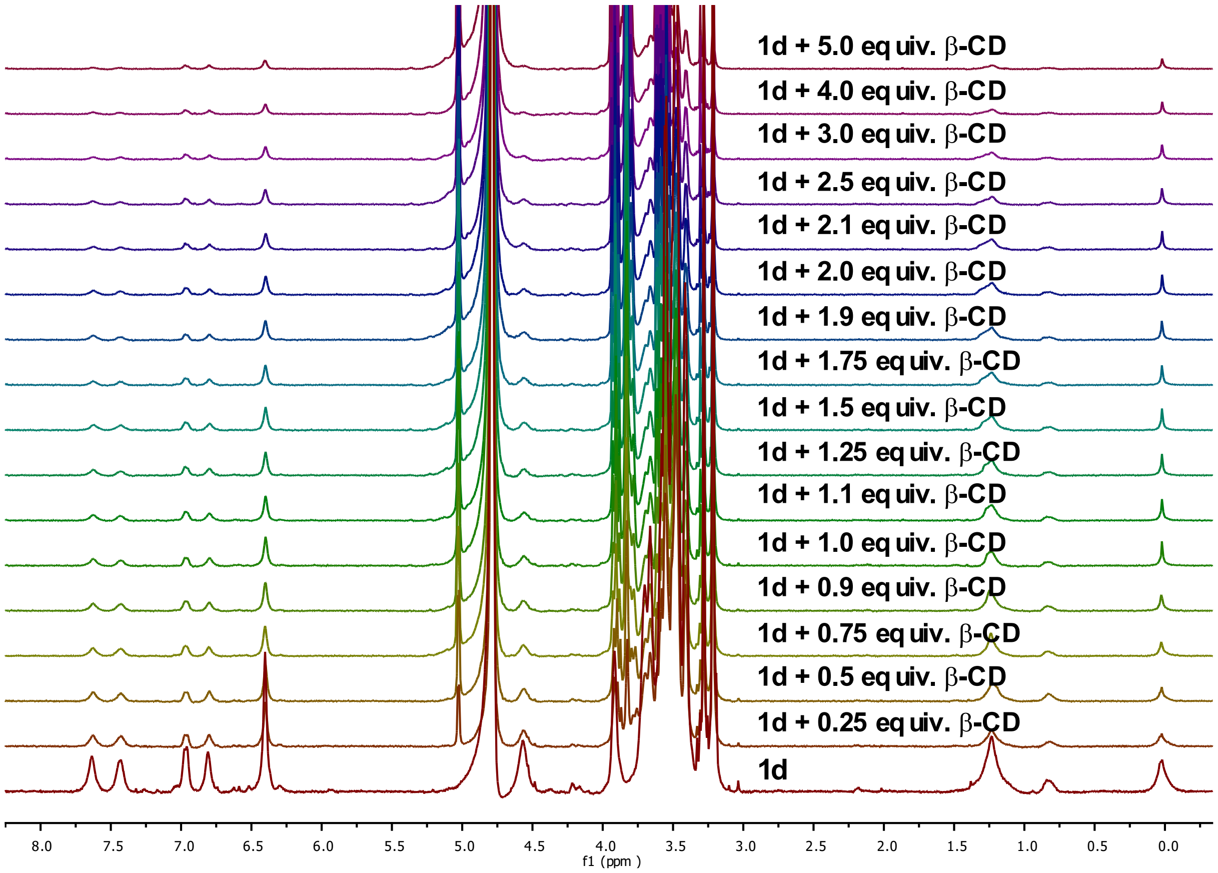


**Figure S14.** Titration of **1d** (2 mM) with increasing amounts of β-CD (bottom to top) $^1$H NMR spectrum (400 MHz, $D_2O$ with NaOD, 298 K).

# S7. ADDITIONAL SWITCHING EXPERIMENTAL DATA

## S7.1. $^{1}H$-NMR spectra of Diels-Alder products in $D_2O$

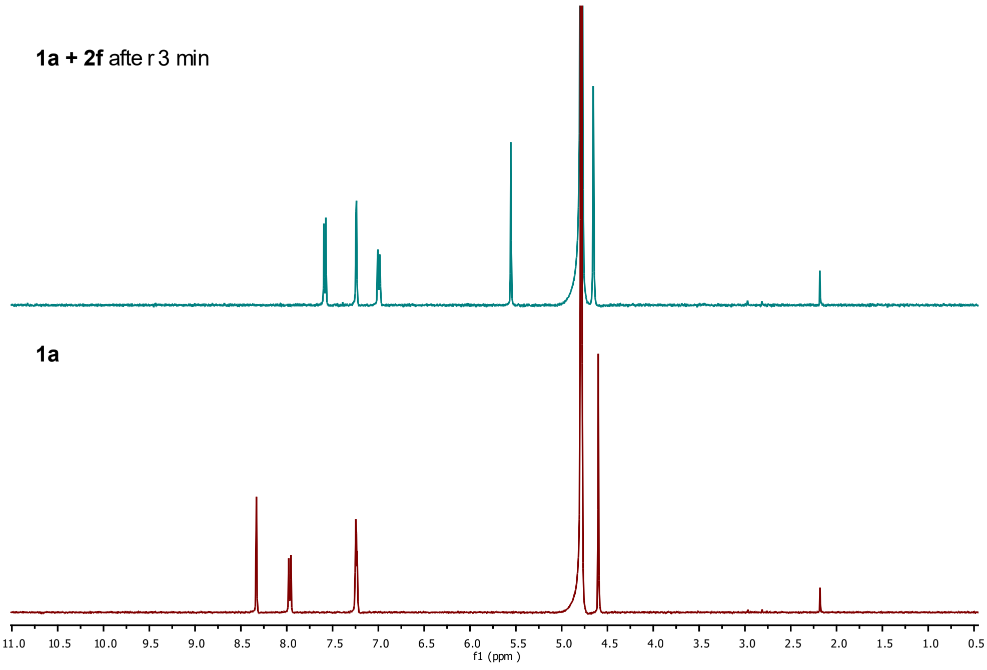


**Figure S15.** Reaction of anthracene **1a** (1 mM) with dienophile **2f** (20 equiv.) after 3 min $^{1}H$ NMR spectrum (400 MHz, $D_2O$ with 8 mM NaOD, 298 K).

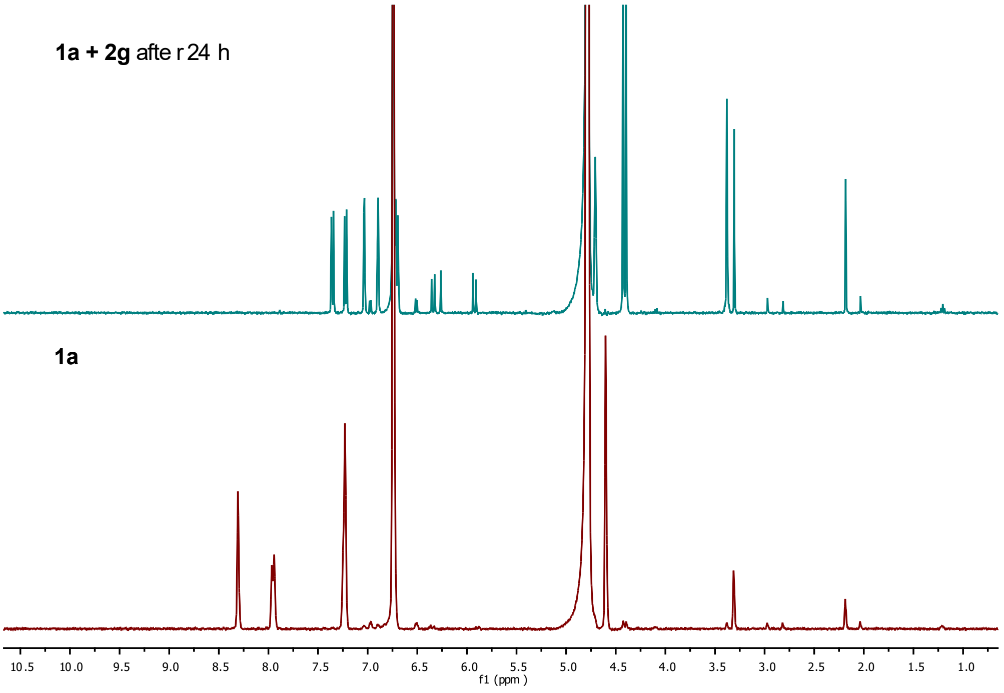


**Figure S16.** Reaction of anthracene **1a** (1 mM) with dienophile **2g** (10 equiv.) after 24 h at 30 °C $^{1}H$ NMR spectrum (400 MHz, $D_2O$ with 8 mM NaOD, 298 K).

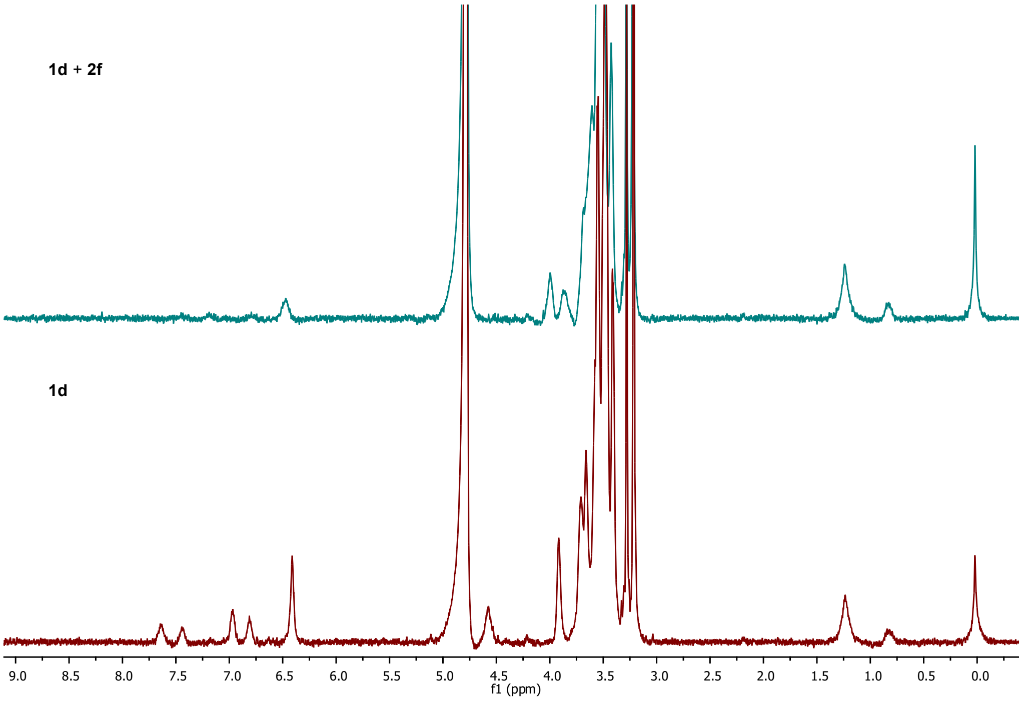


**Figure S17.** Reaction of anthracene **1b** (1 mM) with dienophile **2f** (20 equiv.). $^{1}$H NMR spectrum (400 MHz, $D_2O$ with 8 mM NaOD, 298 K).

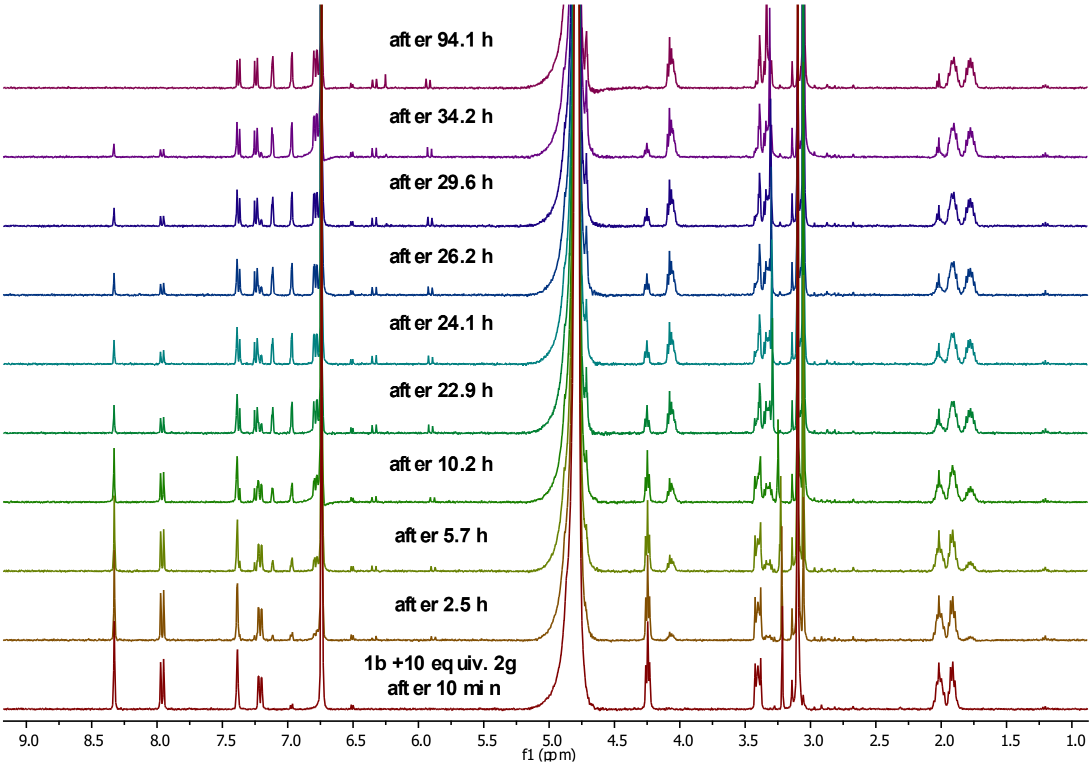


**Figure S18.** Reaction of anthracene **1b** (1 mM) with dienophile **2g** (10 equiv.) over time at RT. $^{1}$H NMR spectrum (400 MHz, $D_2O$ with 8 mM NaOD, 298 K).

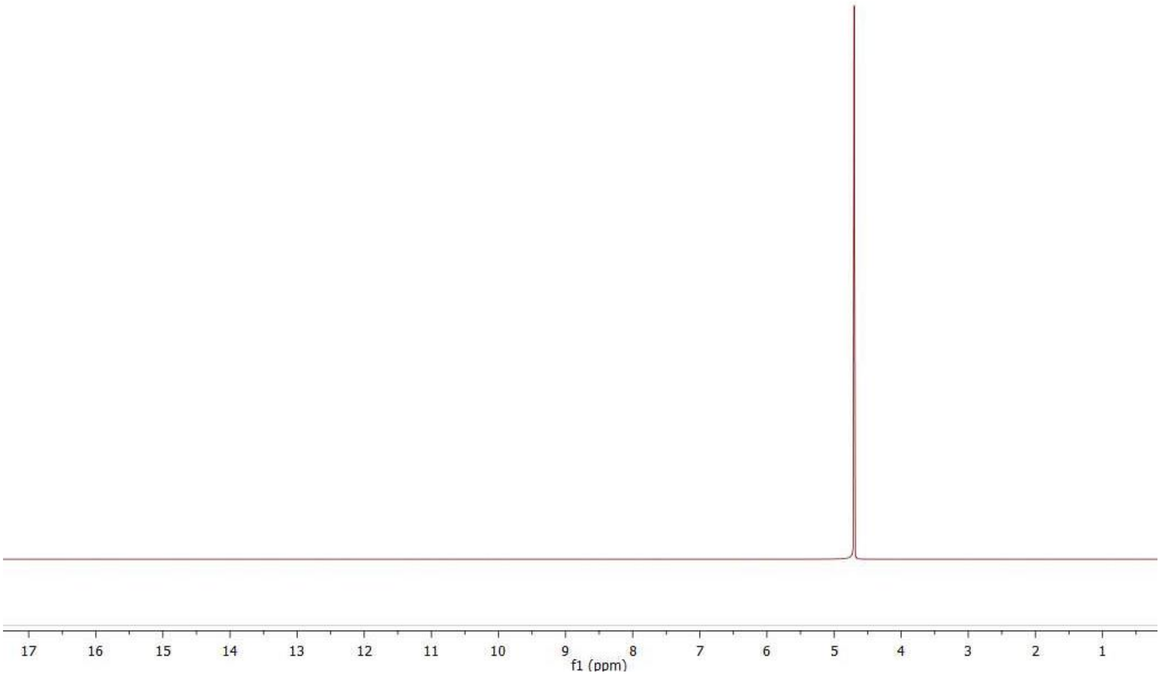


**Figure S19.** $^{1}$H NMR spectrum of TCNE **2f** (400 MHz, $D_2O$, 298 K) for comparison with Figures S15 and S17.

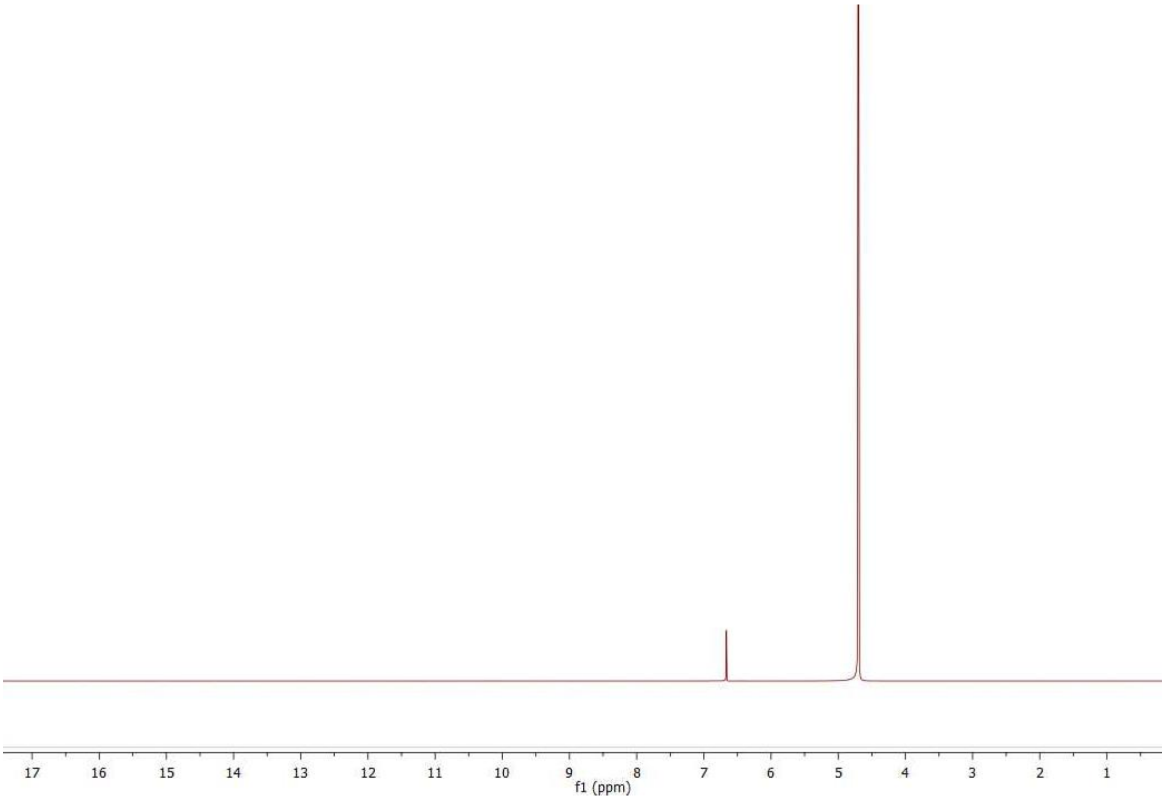


**Figure S20.** $^{1}$H NMR spectrum of maleimide **2g** (400 MHz, $D_2O$, 298 K) for comparison with Figures S16 and S18.

## S7.2. ESI-HRMS spectra of Diels-Alder products

### 1a2f

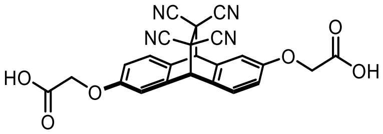


**HRMS** (25% MeOH / 75% $H_2O$+0.1% FA, RT, 4.854 min): *m/z* calcd. for $[C_{24}H_{14}N_4O_6]$: 472.1252 $[M+NH_4]^+$, found: 472.1265; 477.0806 $[M+Na]^+$, found: 477.0801; 493.0545 $[M+K]^+$, found: 493.0574.

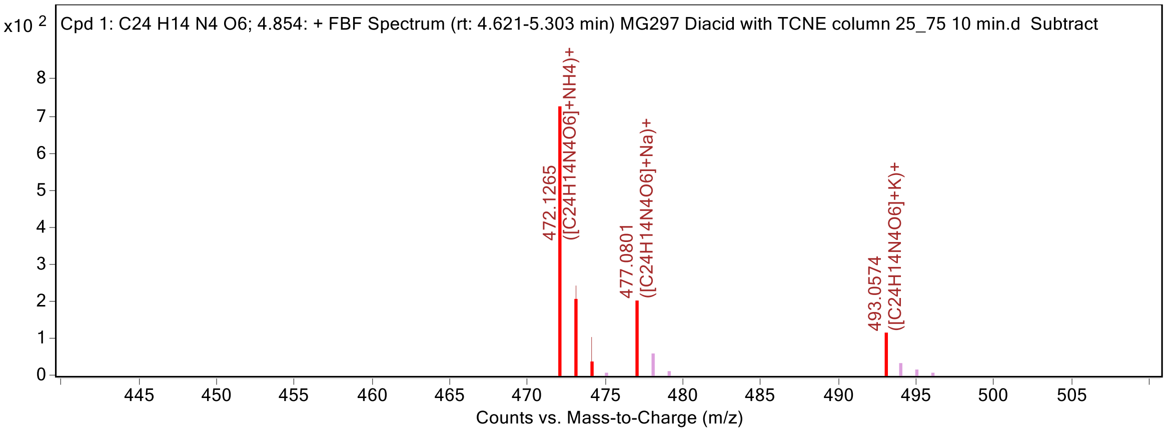


**Figure S21.** High resolution ESI Mass spectrum of **1a2f**.

### 1a2g

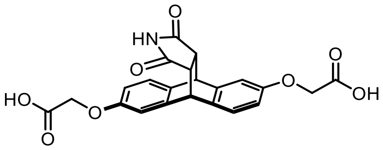


**HRMS** (25% MeOH / 75% $H_2O$+0.1% FA, RT, 3.945 min): *m/z* calcd. for $[C_{22}H_{17}NO_8]$: 424.1027 $[M+H]^+$, found: 424.1040; 441.1292 $[M+NH_4]^+$, found: 441.1317; 446.0846 $[M+Na]^+$, found: 446.0858; 462.0586 $[M+K]^+$, found: 462.0522.

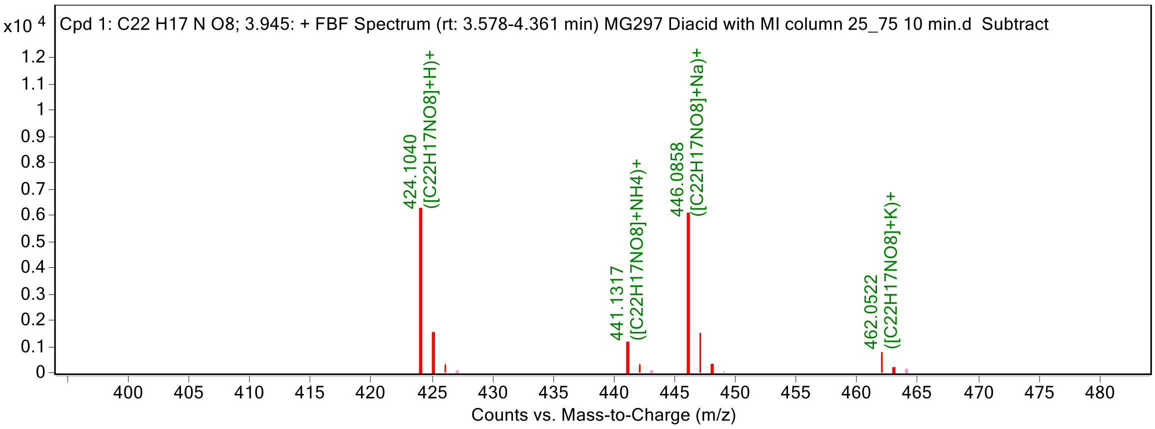


**Figure S22.** High resolution ESI Mass spectrum of **1a2g**.

## 1b2f

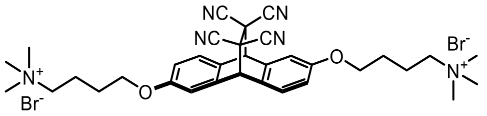


**HRMS** (MeCN): *m/z* calcd. for $[C_{34}H_{42}N_6O_2Br]^+$: 647.2531 $[M+H]^+$, found: 647.2558.

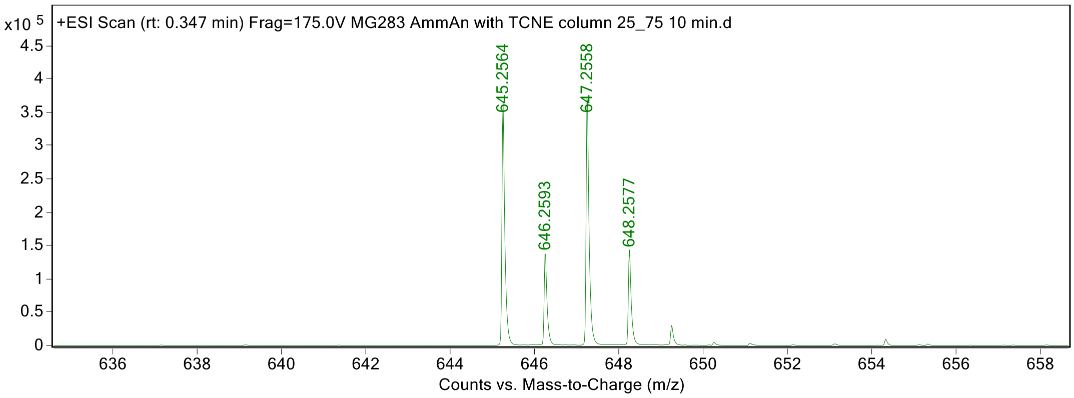


**Figure S23.** High resolution ESI Mass spectrum of **1b2f**.

## 1b2g

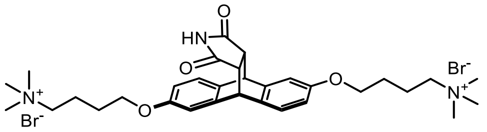


**HRMS** (MeCN): *m/z* calcd. for $[C_{32}H_{45}N_3O_4]^{2+}$: 267.6699 $[M]^{2+}$, found: 267.6720.

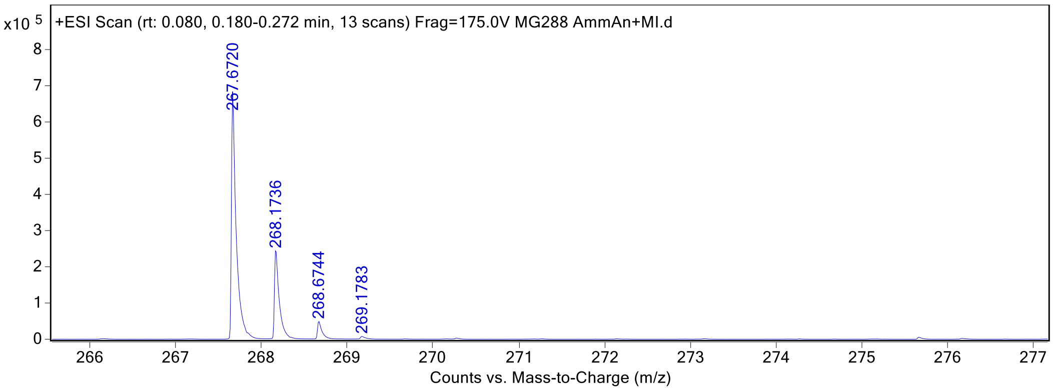


**Figure S24.** High resolution ESI Mass spectrum of **1b2g**.

## S7.3. Switching host-guest chemistry with Diels Alder reaction

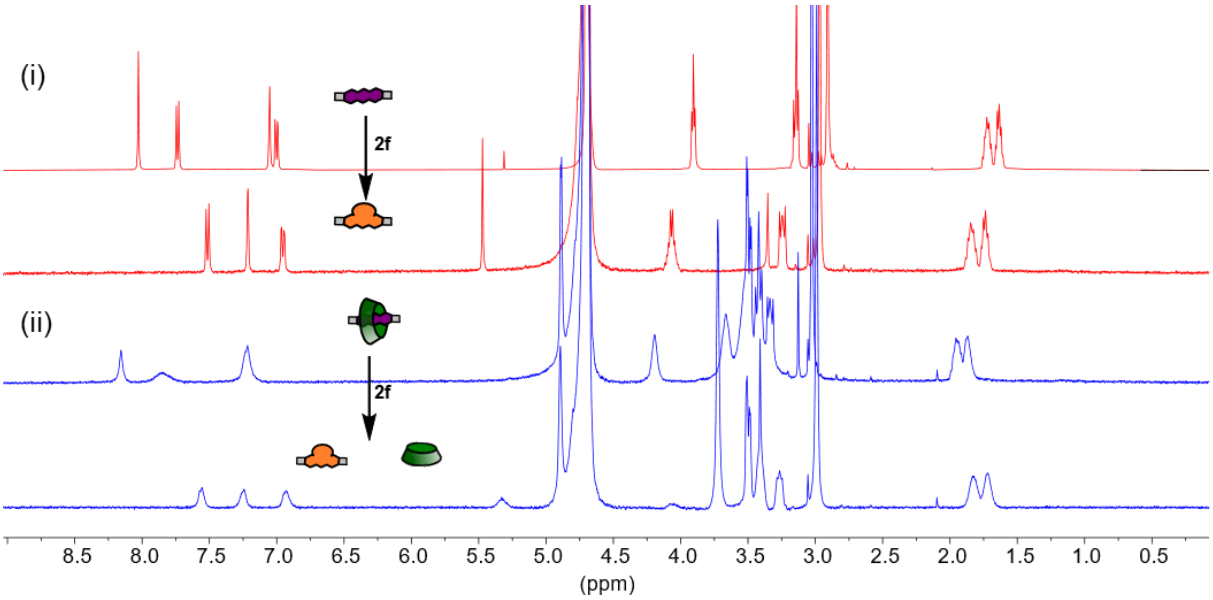


**Figure S25.** $^1$H-NMR spectra (400 MHz, $D_2O$ with 8 mM NaOD, 298 K) of Diels-Alder reactions on **1b**. (i) Reaction of anthracene **1b** (1 mM, top) with dienophile **2f** (20 equiv.) to generate 1b2f (second from top). (ii) Reaction of anthracene-CD complex **1b**•β-CD (1 mM 1b + 1 mM β-CD, second from bottom) with dienophile **2f** (20 equiv.) to generate 1b2f and free β-CD (bottom).

## S7.4. Retro-Diels Alder reactions with DMSO-$d_6$ as scavenger

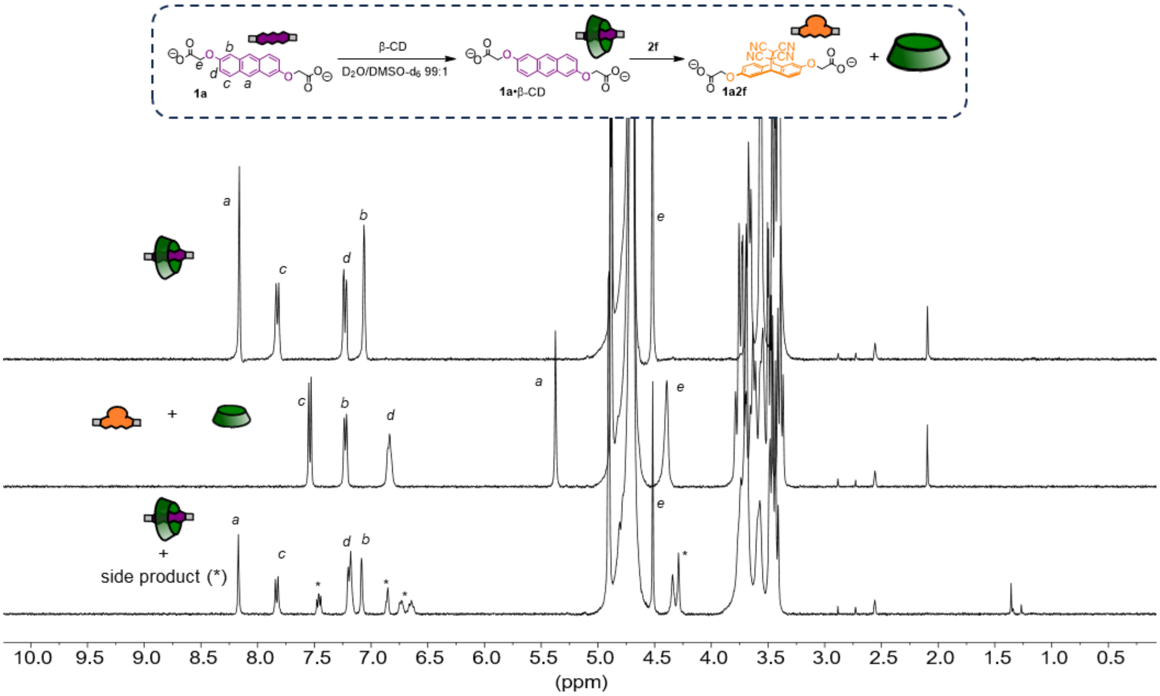


**Figure S26.** Reaction of cycloaddition adduct **1a2f** (1 mM) and **b-CD** (1 equiv.) with DMSO-$d_6$ (1 vol%). The back reaction stops at ~50% even after addition of more DMSO-$d_6$. $^1$H-NMR spectra (400 MHz, $D_2O$/DMSO-$d_6$ 99:1, 298 K) of **1a•**β-CD (1 mM, top), the solution immediately after addition of TCNE **2f** (20 equiv., middle) and again after 48 h (bottom). Note that the identity of the side product/s marked with * in the bottom spectra is currently unknown.

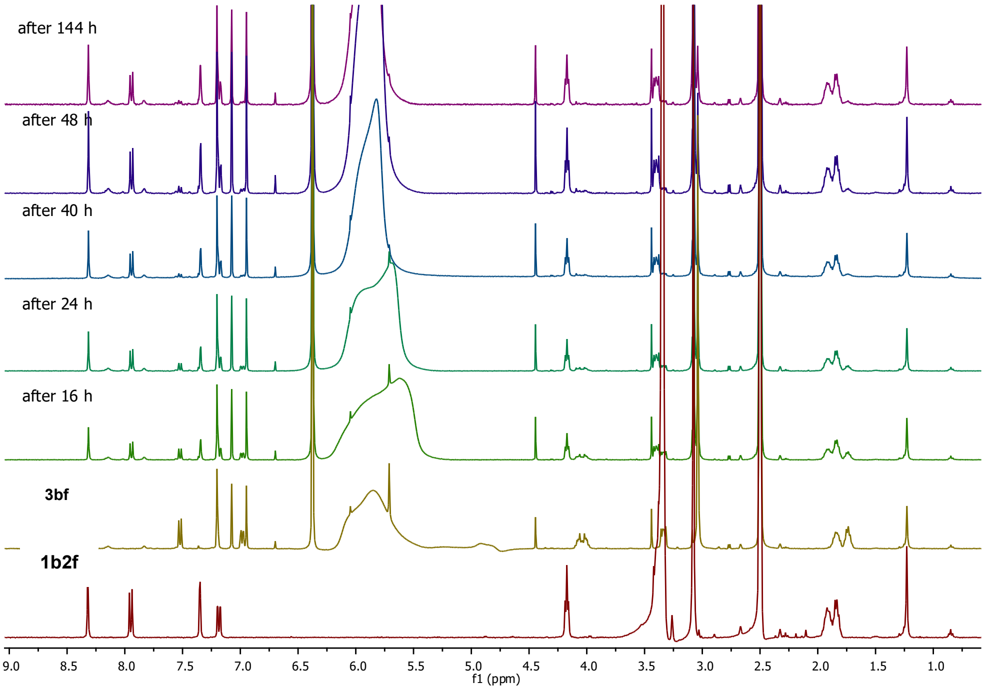


**Figure S27.** Reaction of DA product **1b2f** (1 mM) with DMSO-$d_6$. $^1$H NMR spectrum (400 MHz, DMSO-$d_6$, 298 K.

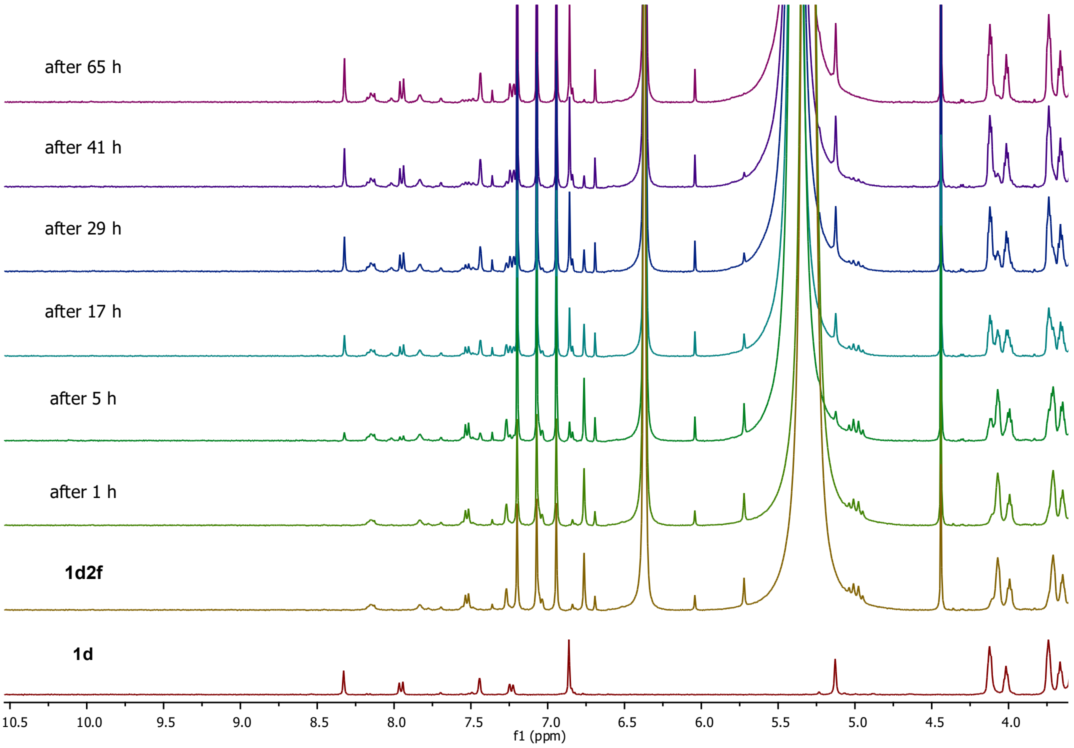


**Figure S28.** Reaction of DA product **1d2f** (1 mM) with DMSO-$d_6$. $^1$H NMR spectrum (400 MHz, DMSO-$d_6$, 298 K).

## S7.5. Retro-Diels Alder reactions with thiol scavengers

Initially, the reaction between dienophile **2f** and various reactive partners (dienes, nucleophiles) was tested in $D_2O$ as shown in Table S2.

**Table S2.** Screening data of reaction between scavenger (2 equiv.) and **2f** (1 mM) as determined by $^1$H NMR (400 MHz, $CDCl_3$, 293 or 313 K).

| Scavenger | T °C | t½ |
|---|---|---|
| furane | 40 | no reaction |
| pyrrole | 40 | no reaction |
| DTT | 20 | 40 h |
| L-cysteine | 20 | 24 h |
| Gluthathione | 20 | 10 h |

The back reaction of **1a2f** using the thiols DTT, GSH and NAcCys in aqueous conditions was next followed by $^1$H NMR spectroscopy. As the scavenging process generates free acid, the reaction needed to be performed in the presence of a base (to keep **1a** and **1a2f** deprotonated and hence soluble). Both HEPES buffer (pH 8) or $D_2O$ with 10 equiv. of $NEt_3$ achieved

satisfactory solubility. Note that the retro-Diels Alder reactions in water are not selective, side products are generated along with the recovered anthracene.

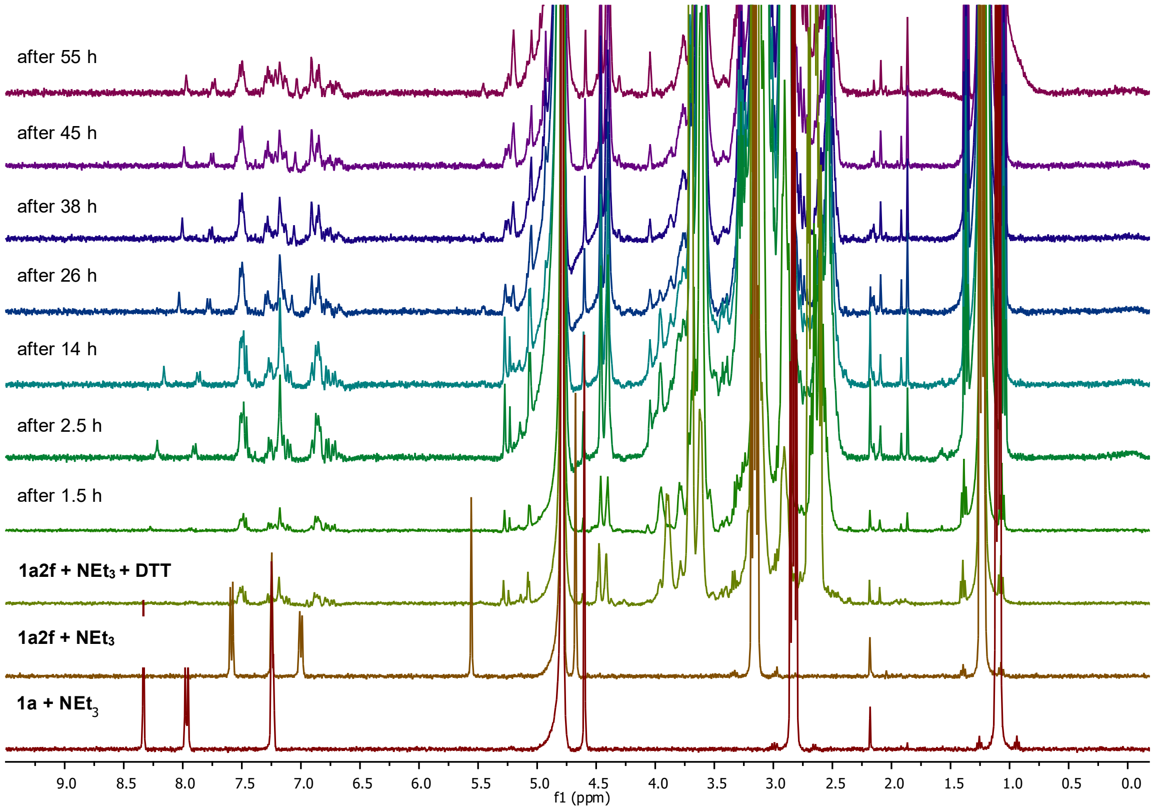


**Figure S29.** Reaction of **1a2f** (1 mM) with DTT (40 equiv.). $^{1}$H NMR spectrum (400 MHz, $D_2O$, 298 K).

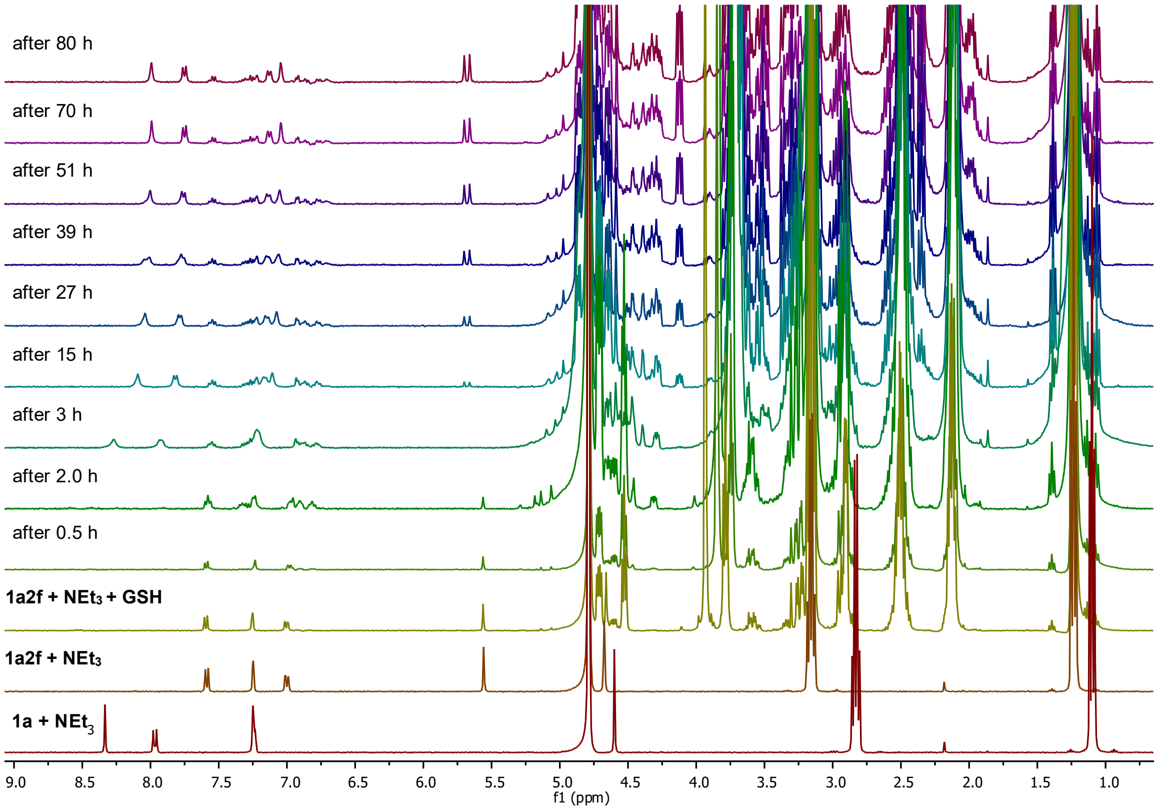


**Figure S30.** Reaction of **1a2f** (1 mM) with GSH (40 equiv.). $^{1}$H NMR spectrum (400 MHz, $D_2O$, 298 K).

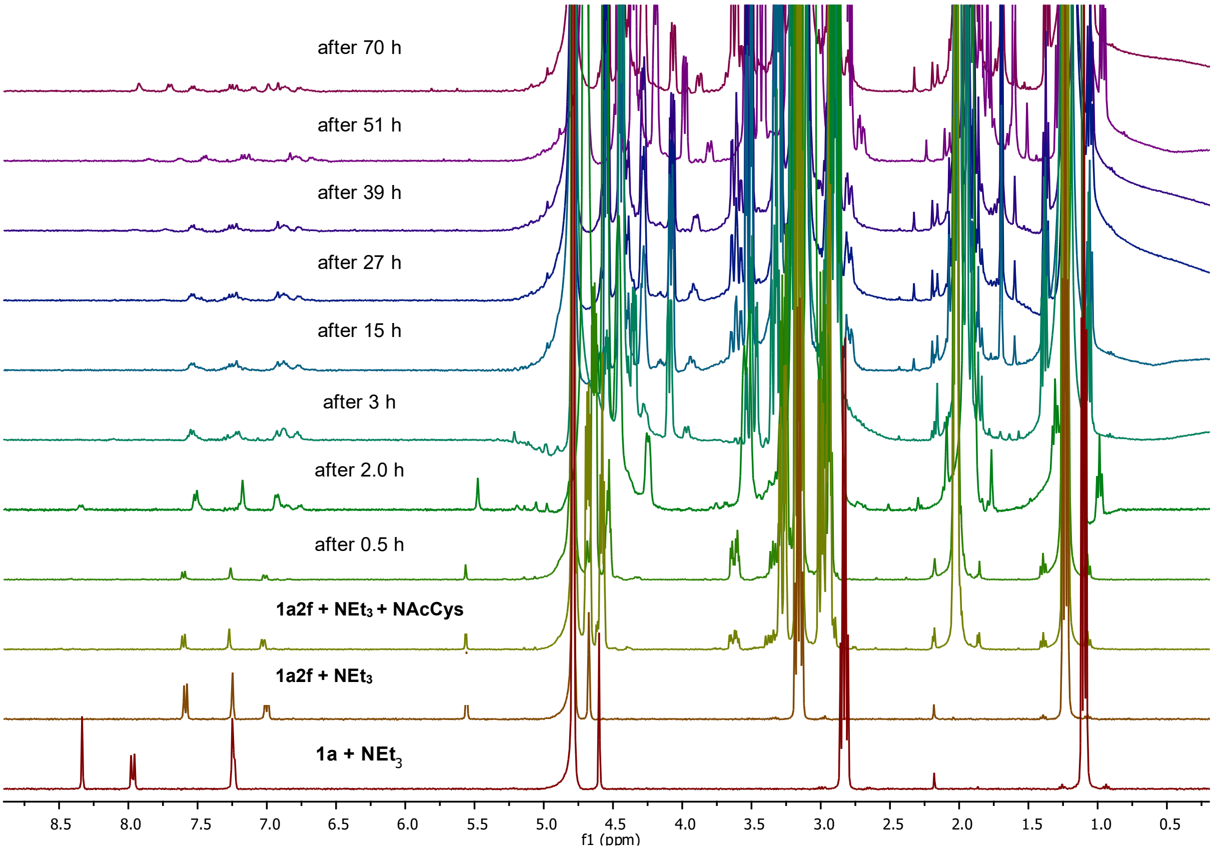


**Figure S31.** Reaction of DA product **1a2f** (1 mM) with NAcCys (40 equiv.). $^{1}H$ NMR spectrum (400 MHz, $D_2O$, 298 K).

The solutions obtained after **1a2f** was reacted with thiols were also investigated qualitatively *in situ* by taking aliquots of the respective reaction mixture and diluting with MeCN. Mass spectra were then collected after elution from a reversed phase column using 20% MeCN / 80% $H_2O$ +0.1 % FA as isocratic eluent. In all cases, the recovered anthracene **1a** was the major observable species (*m/z* calcd. for [$C_{18}H_{14}O_6$]: 327.0863 $[M+H]^+$; found: 327.0854). For the case when GSH was used as scavenger, a mass corresponding to a GSH-**2f** Michael adduct was also observed (20% MeCN / 80% $H_2O$+0.1% FA, RT, 0.3 min): *m/z* calcd. for [$C_{16}H_{17}N_7O_6S$]: 436.1034 $[M+H]^+$, found: 436.1034).

# S8. FLUORESCENCE SPECTROSCOPY

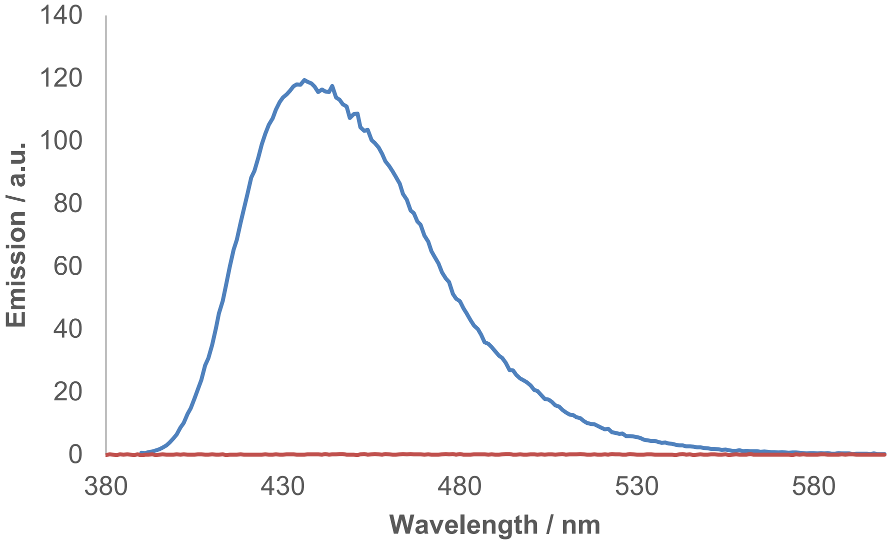


**Figure S32.** Spectra of **1a** (100 μM in $H_2O$ with 8 mM NaOH) before (blue line) and after (red line, overlaps with baseline) addition of **2f**. Emission: $\lambda_{max}$ = 439 nm.

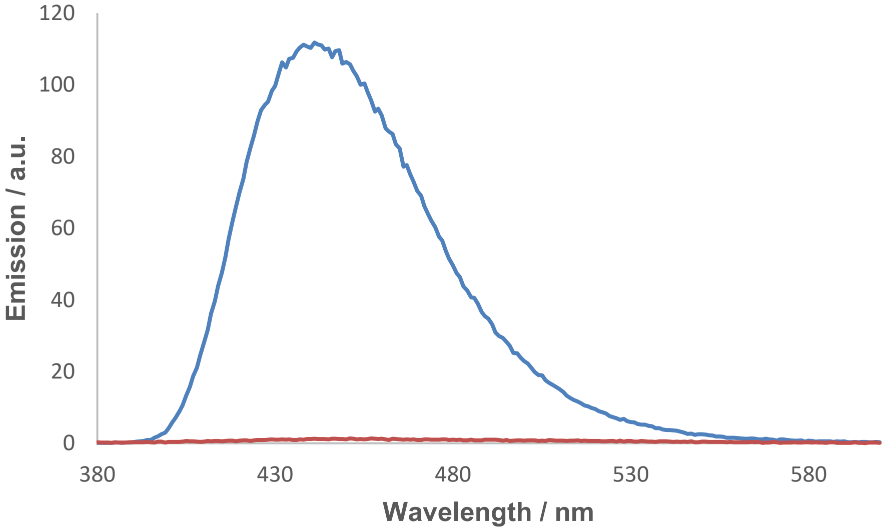


**Figure S33.** Spectra of **1b** (100 μM in $H_2O$) before (blue line) and after (red line, overlaps with baseline) addition of **2f** Emission: $\lambda_{max}$ = 438 nm.

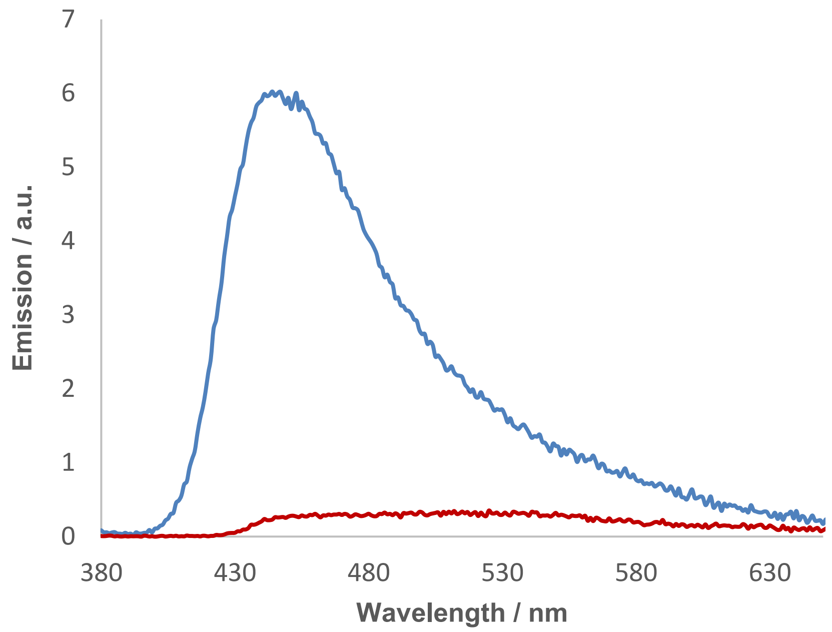


**Figure S34.** Spectra of **1d** (100 μM in $H_2O$) before (blue line) and after (red line) addition of **2f** Emission: $\lambda_{max}$ = 438 nm.

# S9. MOLECULAR MODELLING

Conformational sampling of the guest molecules was carried out using the default iMTD-GC workflow of CREST (version 3.0.1) xTB software (version 6.7.0)[8] at the GFN2-xTB[9] level of theory, using water as implicit solvent (ALPB solvation model).[10] Vibrational frequencies were calculated for the lowest energy structures (within 6 kcal $mol^{-1}$) to identify the most stable conformers by comparing their Total Free Energy. The initial conformer for β-CD host was taken from an X-ray crystal structure (CCDC: 762697)[11] optimised at the GFN2-xTB level of theory (water, ALPB). The xyz coordinates of the optimised minima are provided as additional supplementary files (.zip).

For conformational sampling of the anthracene host-guest complex, a sequence of Minimum Hopping was employed,[12] using a combination of metadynamics, geometry optimisation and frequency calculations. The initial structures were generated by manual placement of the guest into the cavity of the β-CD host using Avogadro (version 1.2.0)[13] and subsequent geometry optimisation at the GFNFF-xTB level of theory. The resulting structure underwent Minimum Hopping (metadynamics detailed input: save=2000; $k_{push}$=0.1 Eh; a = 1.2 $Bohr^{-1}$) with an implicit water solvation model (APLB) at the GFN2-xTB level of theory. Due to the host-guest complex being a non-covalently bound supramolecular complex, metadynamics simulations were carried out under confinement in a sphere of repulsive potential (wall potential=logfermi, sphere: auto, all) aligned with the centre of mass aligned to that of the complex ($cma). The resulting ensemble was reoptimised at the GFN2-xTB level of theory, and then the lowest energy structures (within 6 kcal $mol^{-1}$) were submitted to vibrational frequency calculations (GFN2-xTB) from which the local minimum could be identified based on Total Free Energy.

Metadynamics simulations were carried out on the host-guest complexes with the Diels-Alder adducts, under the same constraints (save=2000; $k_{push}$=0.1 Eh; a = 1.2 $Bohr^{-1}$; wall potential=logfermi, sphere=auto; $cma), which – due to the additional steric hindrance and non-planar configuration of the Diels-Alder adducts – did not remain inside the cavity of the β-CD host. Therefore, local minima for the host-guest complexes with Diels-Alder adducts were not calculated. Movies of each of the metadynamics trajectories are supplied as additional supplementary files (.gif). This workflow was also employed to verify if a guest extremity was too bulky to allow threading, i.e. in the case of compound **1d** and β-CD where we computationally observed conformations prone to slippage.

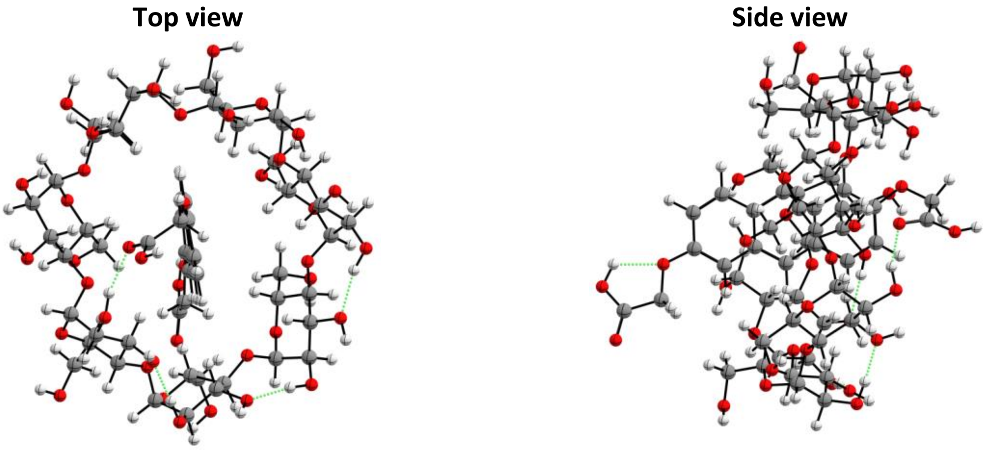


**Figure S35**. Local minimum of the β-CD•**1a** host-guest complex calculated at the GFN2-xTB level using water as implicit solvent (ALPB).

## S10. NMR SPECTRA OF NEW COMPOUNDS

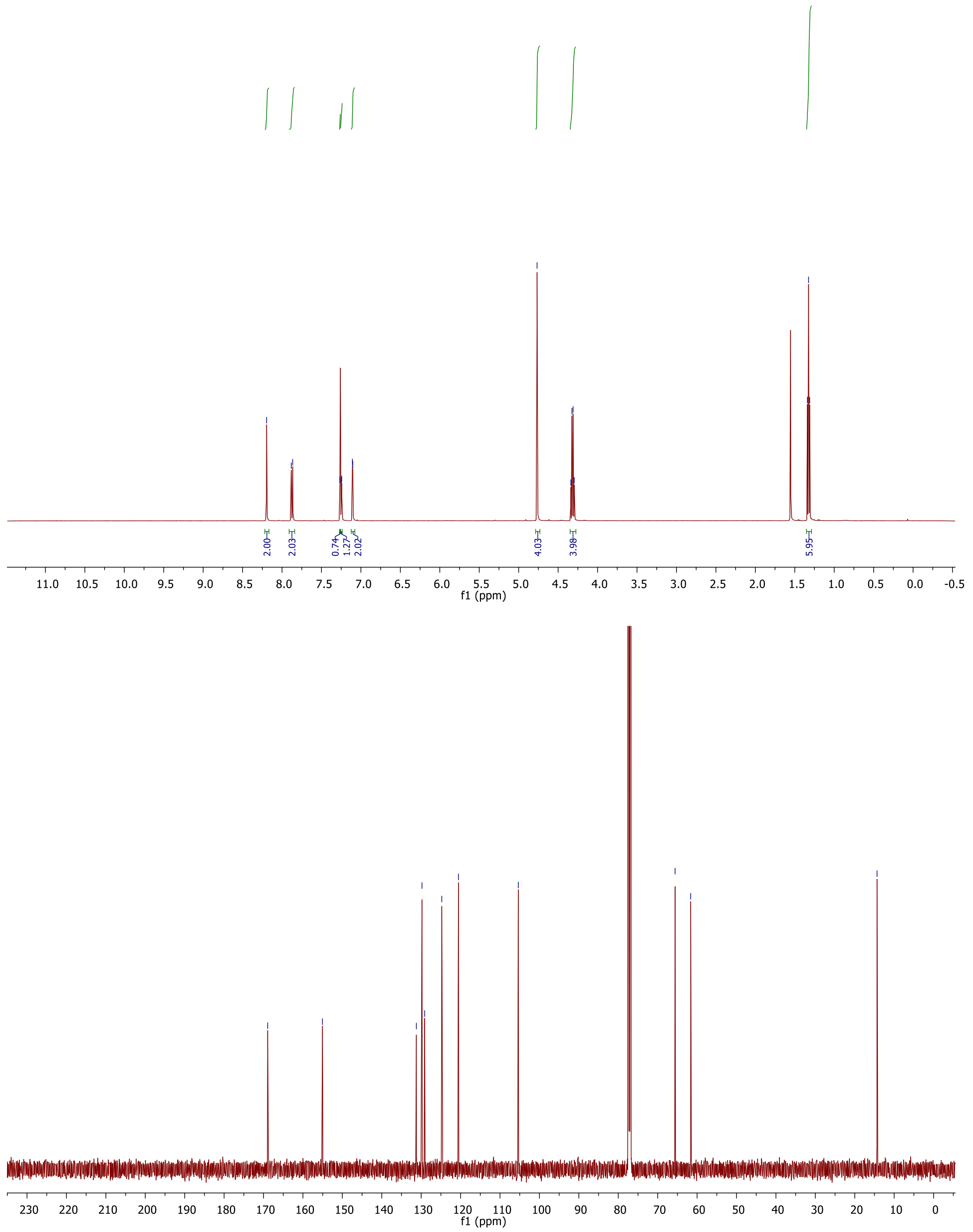


**Figure S36.** $^{1}H$ (top) and $^{13}C$ (bottom) NMR spectrum (500/126 MHz, $CDCl_3$, 298 K) of **S2**.

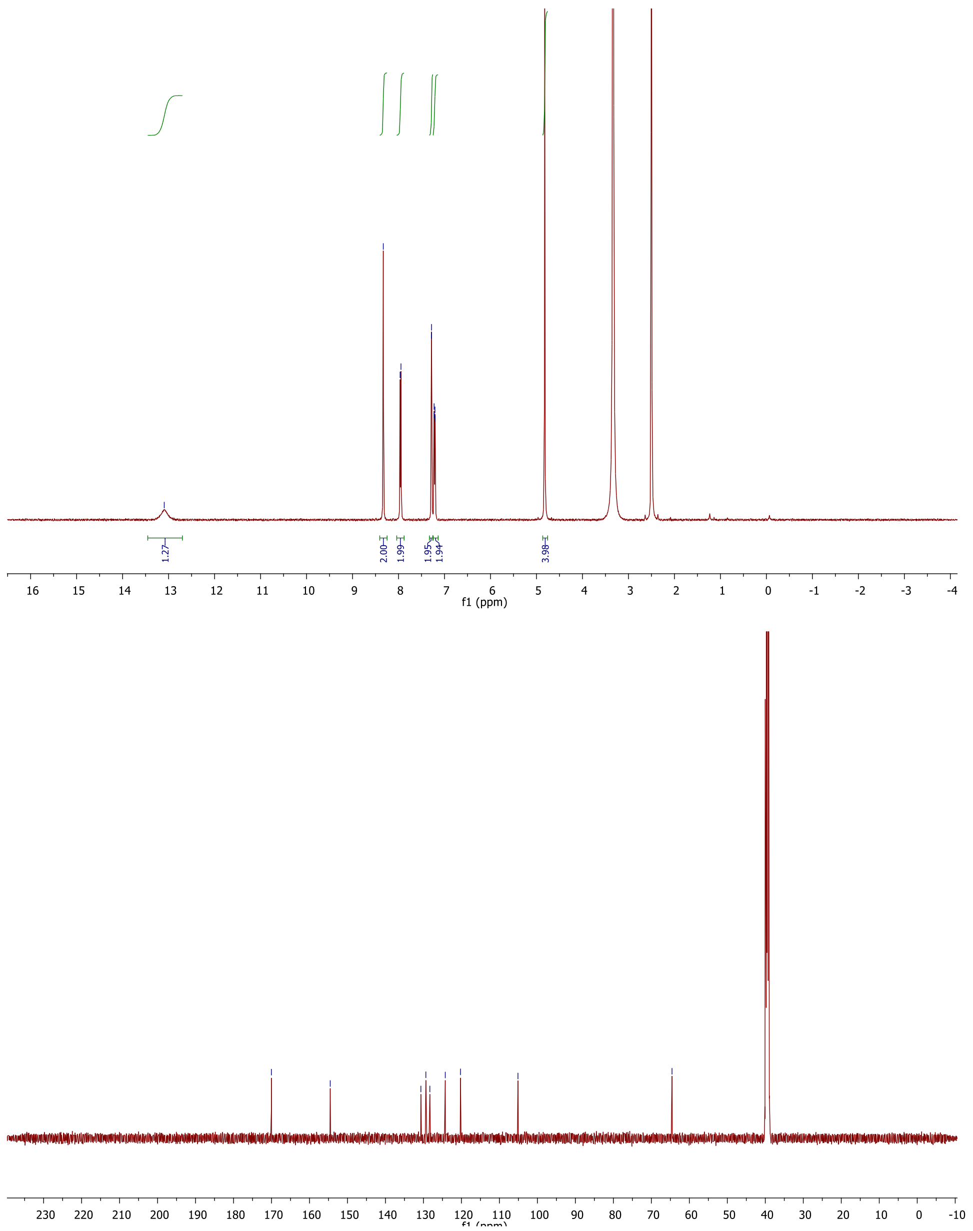


**Figure S37.** $^{1}H$ (top) and $^{13}C$ (bottom) NMR spectrum (500/126 MHz, DMSO-d6, 298 K) of **1a**.

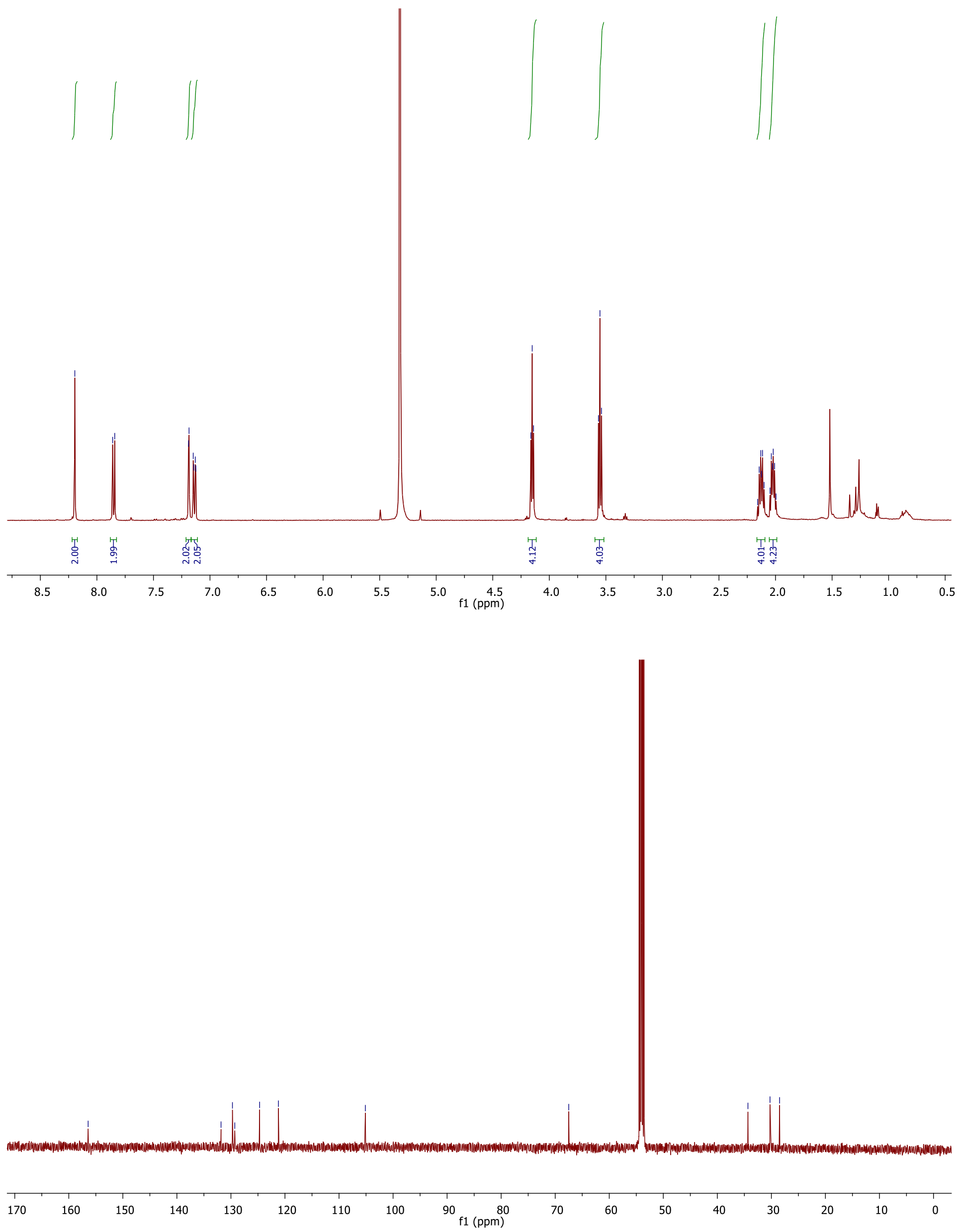


**Figure S38.** $^{1}$H (top) and $^{13}$C (bottom) NMR spectrum (500/126 MHz, $CD_2Cl_2$, 298 K) of **S3**.

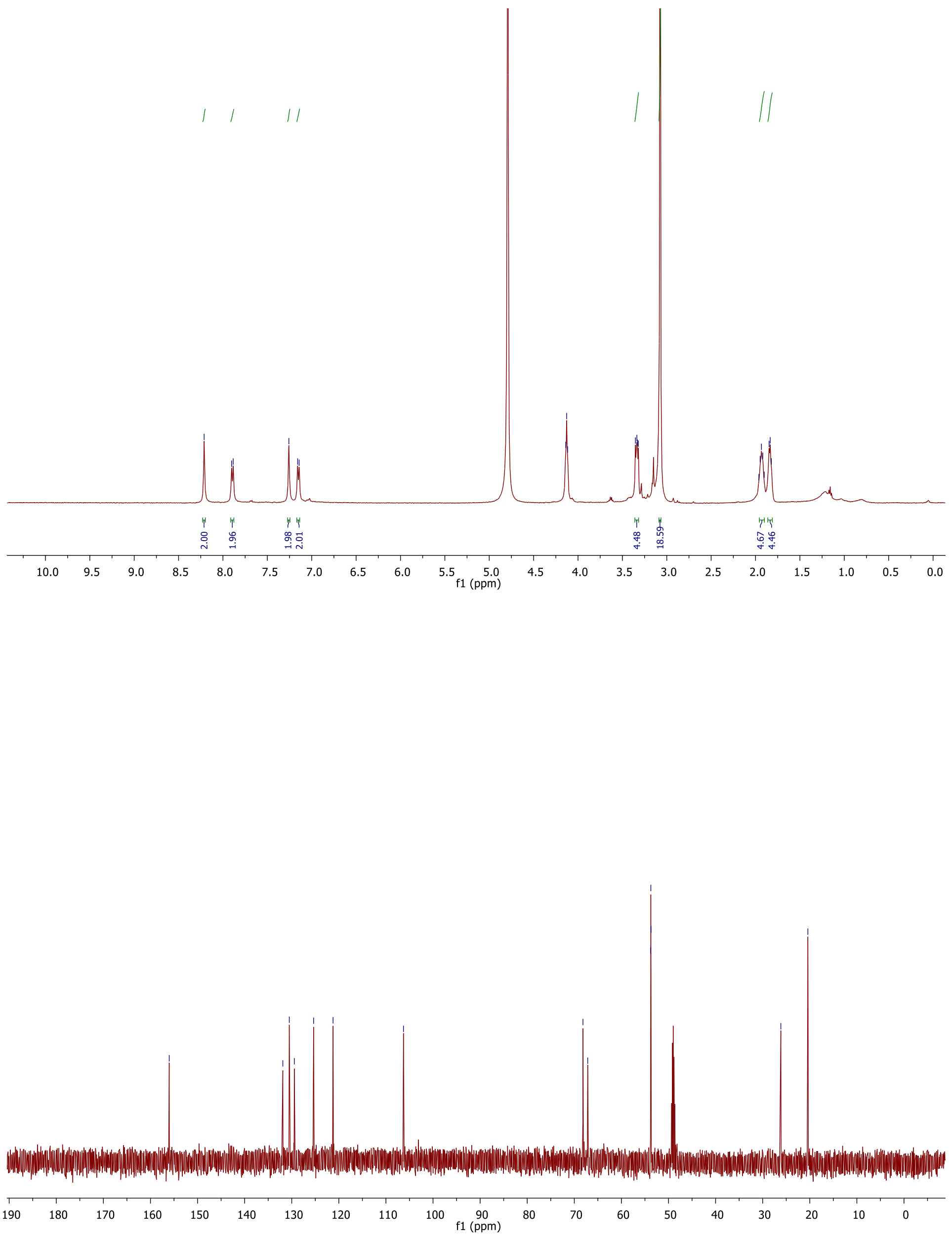


**Figure S39.** $^{1}H$ (top) and $^{13}C$ (bottom) NMR spectrum (500/126 MHz, $D_2O$+MeOD-*d*$_4$, 298 K) of **1b**.

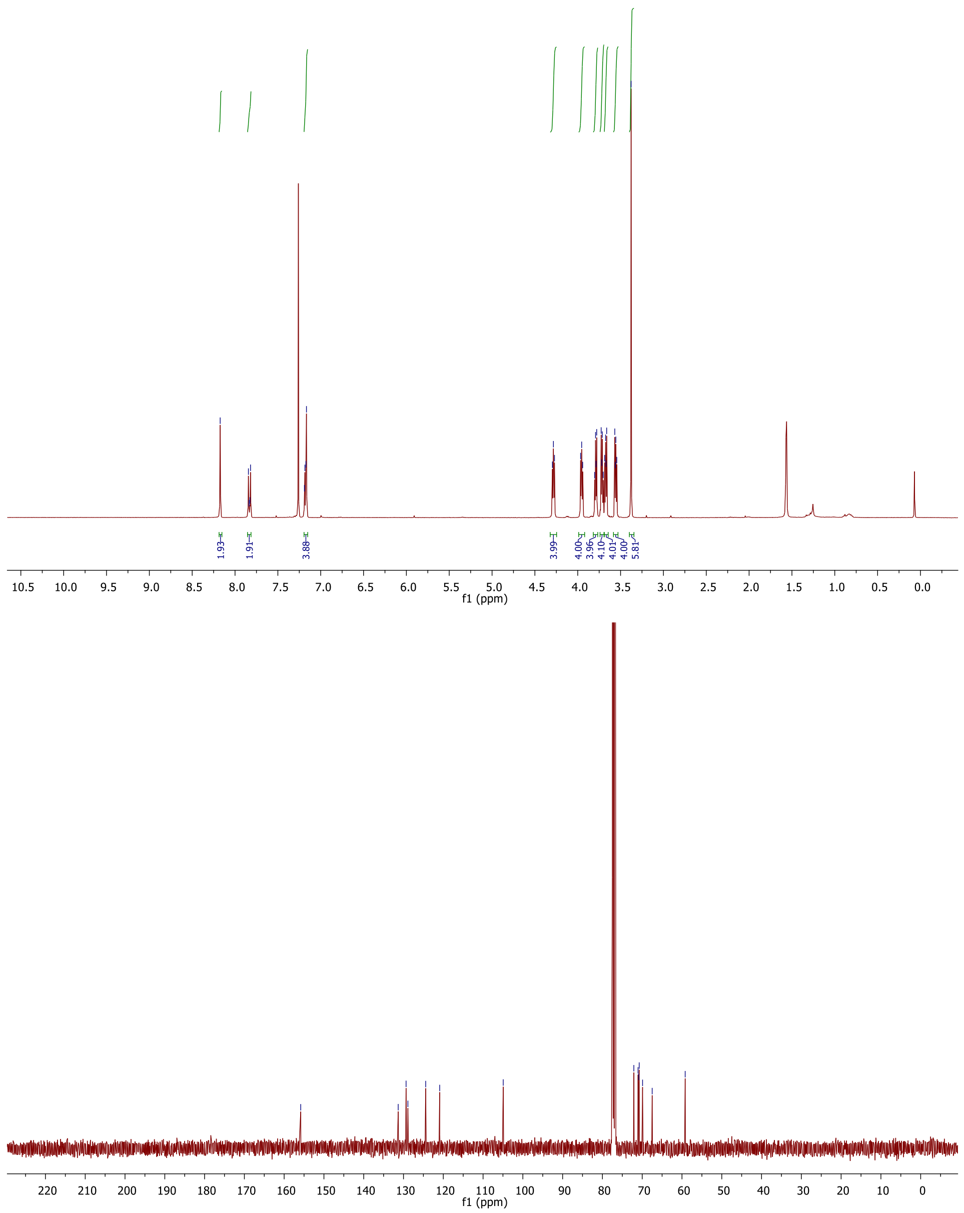


**Figure S40.** $^{1}$H (top) and $^{13}$C (bottom) NMR spectrum (400/101 MHz, $CDCl_3$, 298 K) of **1c**.

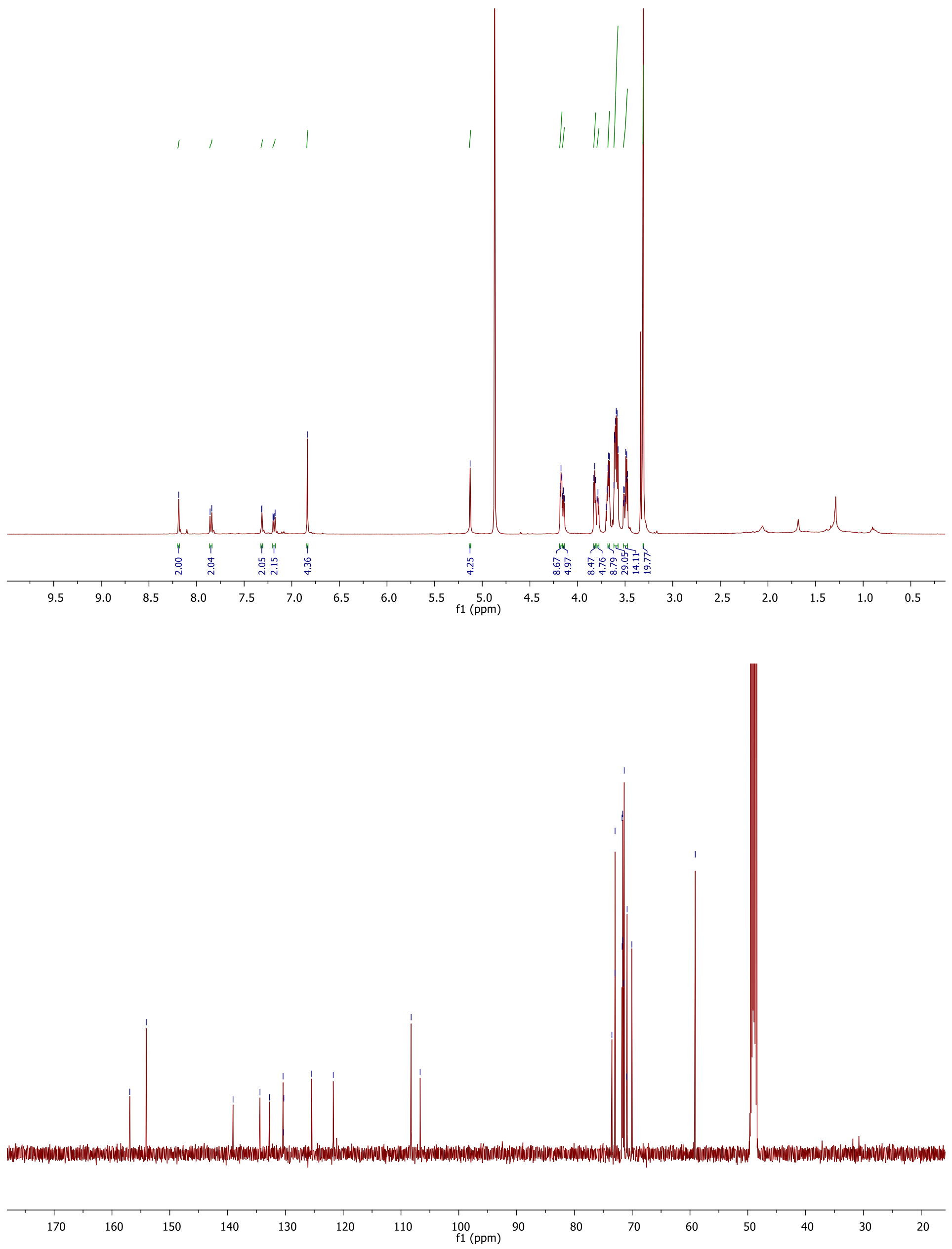


**Figure S41.** $^{1}H$ (top) and $^{13}C$ (bottom) NMR spectrum (500/126 MHz, MeOD, 298 K) of **1d**.

## S11. MASS SPECTRA OF NEW COMPOUNDS

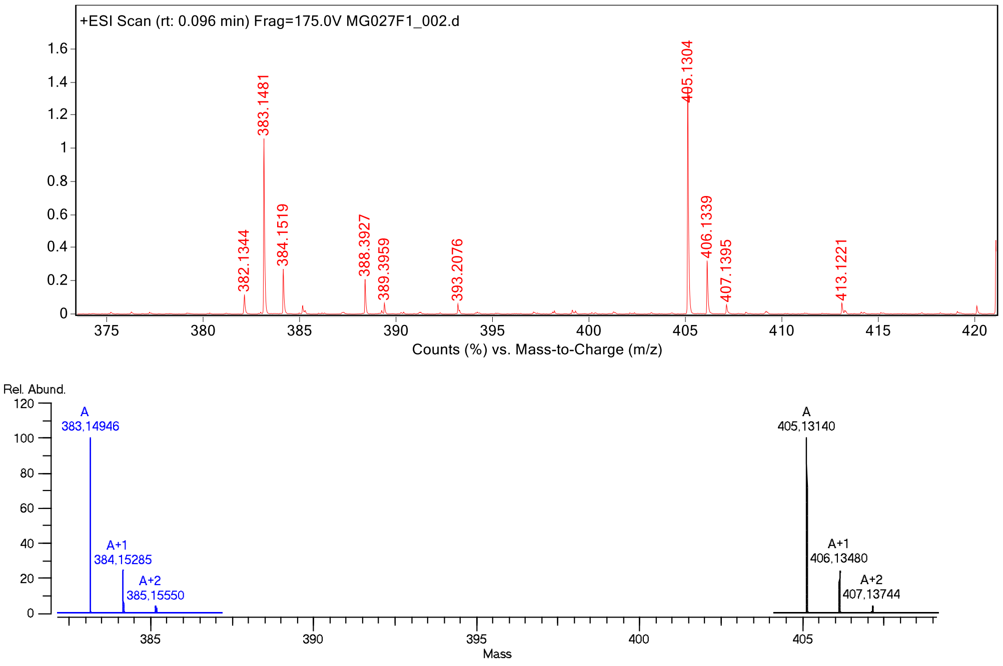


**Figure S42.** High resolution ESI Mass spectrum (MeCN) of **S2**.

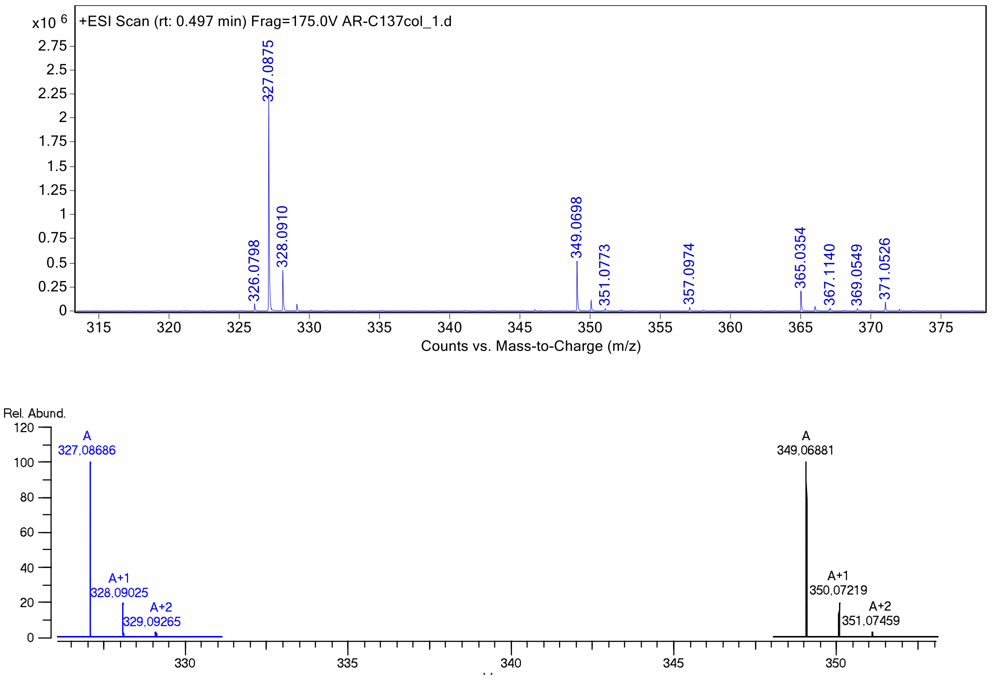


**Figure S43.** High resolution ESI Mass spectrum (MeCN) of **1a**.

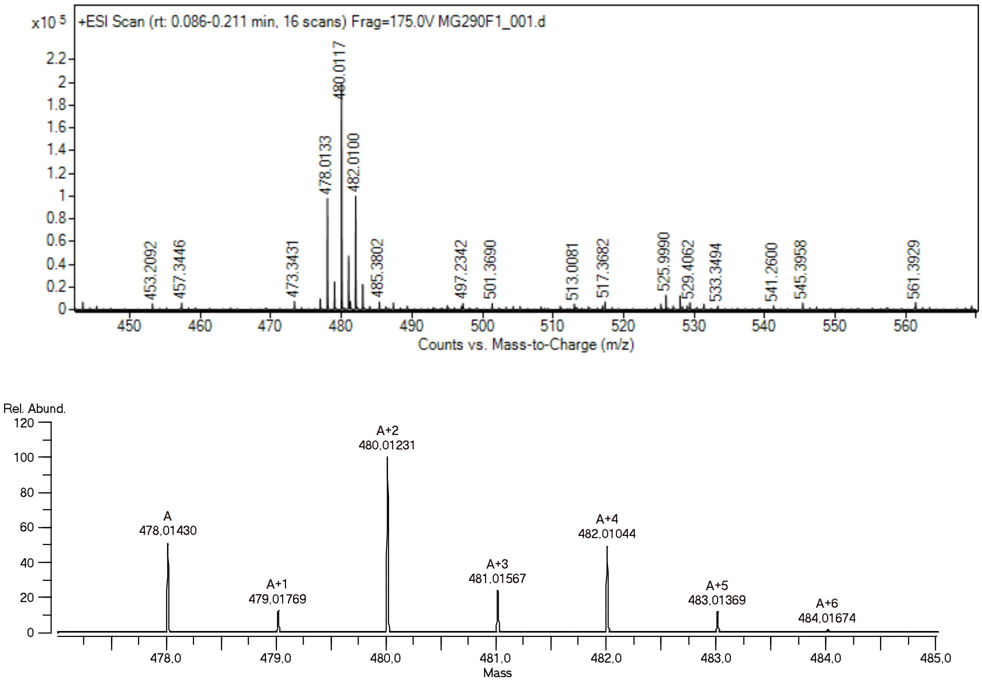


**Figure S44.** High resolution ESI Mass spectrum (MeCN) of **S3**.

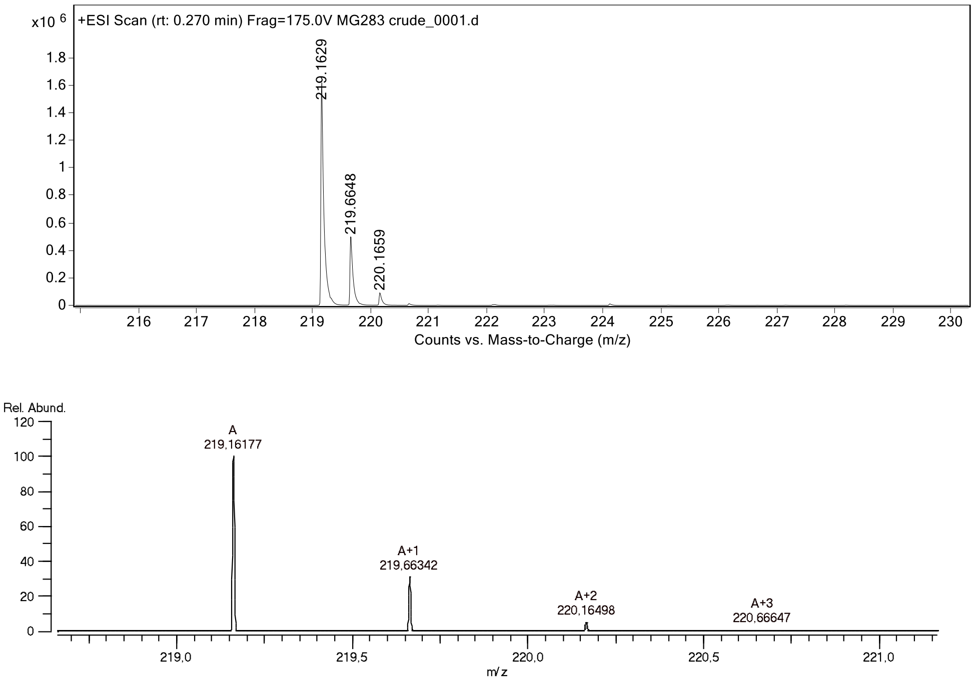


**Figure S45.** High resolution ESI Mass spectrum (MeCN) of **1b**.

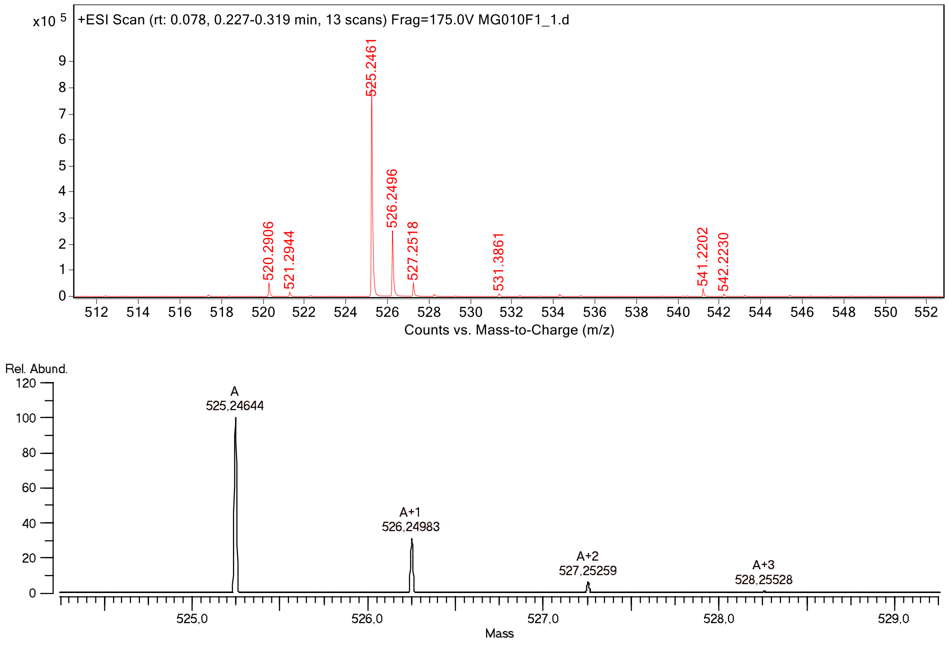


**Figure S46.** High resolution ESI Mass spectrum (MeOH) of **1c**.

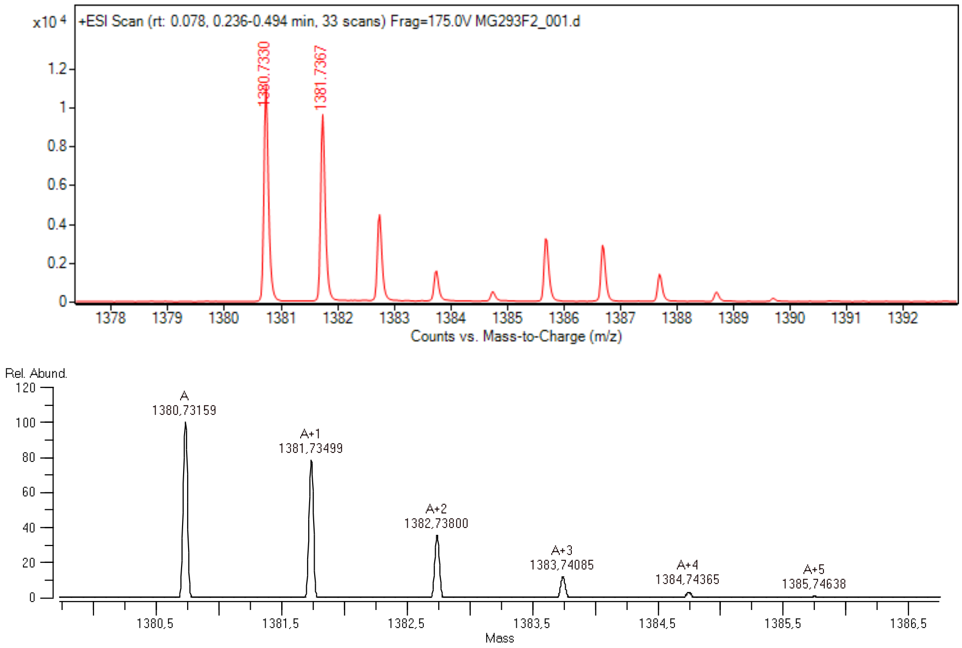


**Figure S47.** High resolution ESI Mass spectrum (MeCN) of **1d**.

# S12. REFERENCES


[1] P. T. Corbett, J. K. M. Sanders, S. Otto, *Chem. Eur. J.* **2008**, *14*, 2153–2166.

[2] E. J. Mitchell, A. J. Beecroft, J. Martin, S. Thompson, I. Marques, V. Félix, P. D. Beer, *Angew. Chem. Int. Ed*. **2021**, *60*, 24048–24053.

[3] S. Kohmoto, E. Mori, K. Kishikawa, *J. Am. Chem. Soc.* **2007**, *129*, 13364–13365.

[4] W. Tang, S.-C. Ng, *Nat. Protoc*. **2008**, *3*, 691–697.

[5] D. B. Hibbert, P.Thordarson, *Chem. Commun.*, **2016**, *52*, 12792–12805.

[6] P. Thordarson, *Chem. Soc. Rev.*, **2011**, *40*, 1305–1323.

[7] T. Tamaki, T. Kokubu, K. Ichimura, *Tetrahedron*, **1987**, *43*, 1485–1494.

[8] C. Bannwarth, E. Caldeweyher, S. Ehlert, A. Hansen, P. Pracht, J. Seibert, S. Spicher, S. Grimme, *WIREs Computational Molecular Science*, **2021**, *11*, e1493.

[9] C. Bannwarth, S. Ehlert, S. Grimme, *J. Chem. Theory Comput*. **2019**, *15*, 1652–1671.

[10] S. Ehlert, M. Stahn, S. Spicher, S. Grimme, *J. Chem. Theory Comput*. **2021**, *17*, 4250–4261.

[11] A. I. Ramos, T. M. Braga, P. Silva, J. A. Fernandes, P. Ribeiro-Claro, M. de F. S. Lopes, F. A. A. Paz, S. S. Braga, *CrystEngComm*, **2013**, *15*, 2822–2834.

[12] A. Závodná, P. Janovský, V. Kolařík, J. S. Ward, Z. Prucková, M. Rouchal, K. Rissanen, R. Vicha, *Chem. Sci.* **2025**,*16*, 83–89

[13] M. D. Hanwell, D. E. Curtis, D. C. Lonie, T. Vandermeersch, E. Zurek, G. R. Hutchison, *J. Cheminf*. **2012**, *4*, 17.
