# Supplementary material for: Controlling cyclodextrin host-guest complexation in water with dynamic pericyclic chemistry
Source: Commun Chem. 2025 Dec 26;9:51. doi: 10.1038/s42004-025-01858-8 (PMC12847988; doi:10.1038/s42004-025-01858-8)
Supplement: Supplementary file 3 — Description of Additional Supplementary Files [file 42004_2025_1858_MOESM3_ESM.pdf]

Description of Additional Supplementary Files:

File: Supplementary Data 1

Description: xyz coordinations for molecular dynamics simulations.

File: Supplementary Data 2

Description: Source data file with raw data for main manuscript figures

File: Supplementary Data 3

Description: NMR spectra of new compounds

File: Supplementary Movie 1

Description: Snapshot of molecular metadynamics simulation of Diels-Alder adduct 1a2f and β-CD, starting from a forced host-guest complex.

File: Supplementary Movie 2

Description: Snapshot of molecular metadynamics simulation of Diels-Alder adduct 1a2g and β-CD, starting from a forced host-guest complex.

File: Supplementary Movie 3

Description: Snapshot of molecular metadynamics simulation of anthracene 1a and β-CD, starting from a forced host-guest complex.
